# The porcine odorant-binding protein as molecular probe for benzene detection

**DOI:** 10.1371/journal.pone.0202630

**Published:** 2018-09-05

**Authors:** Alessandro Capo, Angela Pennacchio, Antonio Varriale, Sabato D'Auria, Maria Staiano

**Affiliations:** Institute of Food Science, Consiglio Nazionale delle Ricerche, Avellino, Italy; Purdue University, UNITED STATES

## Abstract

In recent years, air pollution has been a subject of great scientific and public interests for the strong impact on human health. Air pollution is due to the presence in the atmosphere of polluting substances, such as carbon monoxide, sulfur and nitrogen oxides, particulates and volatile organic compounds (VOCs), derived predominantly from various combustion processes. Benzene is a VOC belonging to group-I carcinogens with a toxicity widely demonstrated. The emission limit values and the daily exposure time to benzene (TLV-TWA) are 5μg/m^3^ (0.00157 ppm) and 1.6mg/m^3^ (0.5 ppm), respectively. Currently, expensive and time-consuming analytical methods are used for detection of benzene. These methods require to perform a few preliminary steps such as sampling, and matrices pre-treatments. In addition, it is also needed the support of specialized personnel. Recently, single-walled carbon nanotube (SWNTs) gas sensors with a limit detection (LOD) of 20 ppm were developed for benzene detection. Other innovative bioassay, called bio-report systems, were proposed. They use a whole cell (*Pseudomona putida* or *Escherichia coli)* as molecular recognition element and exhibit a LOD of about 10 μM. Here, we report on the design of a highly sensitive fluorescence assay for monitoring atmospheric level of benzene. For this purpose, we used as molecular recognition element the porcine odorant-binding protein (pOBP). 1-Aminoanthracene was selected as extrinsic fluorescence probe for designing a competitive fluorescence resonance energy transfer (FRET) assay for benzene detection. The detection limit of our assay was 3.9μg/m^3^, a value lower than the actual emission limit value of benzene as regulated by European law.

## Introduction

In recent decades, the degradation of air-water quality has produced dangerous effects on the natural environments and human health, becoming one of main global alarms. The principal cause of air-water degradation is the presence in environment of organic compounds that are products from the petroleum and pesticide industries. For example, manmade releases of organic compounds derive from evaporative petrol storage, motor vehicles, cigarettes smoke and natural sources (like forest fires and volcanic emissions). Air pollution is the main environmental cause of death in Europe [1, 2]. In particular, heart diseases and stroke are the most common causes of death associate to air pollution [2]. Furthermore, air pollution increases the incidence of a wide range of diseases (e.g. respiratory and cardiovascular diseases and cancer), with both long- and short-term health effects (fertility, pregnancy, new-born and children) [3, 4].

The volatile organic compounds (VOCs), in particular, have high vapor pressure values and they are available as gas at ambient pressure and temperature values.

A group of VOCs, called BTEX, (benzene, toluene, ethyl-benzene and xylene isomers), generally is monitored for both ambient and industrial applications as well as health and safety claims.

Among BTEX, benzene, a volatile, colorless and odorant liquid, is considered the most dangerous component [5–7]. Due to its volatile nature, the main route of human exposition to benzene is represented by inhalation [8, 9]. The threshold limit value -time weight average (TLV-TWA) is 1.6 mg /m^3^ (0.5 ppm), while the atmospheric limit for safety of human health is 5μg/m^3^ (0.00157 ppm) [10].

The current techniques used to monitor atmospheric levels of BTEX include the use of analytical approaches such as gas-chromatography coupled to mass spectrometry. An alternative method for BTEX detection is represented by active and automatic sampling coupled with flame ionization detector gas-chromatography. This method operates an off-line monitoring using the samples collection and analysis performed by specialized laboratories. This results in an increase of time and costs of the analyses [11, 12].

New sensing approaches to detect BTEX have been explored focusing on user-friendly features and outdoor use. For example, for air monitoring applications, an innovative passive sampling device based on processes such as diffusion and permeation was developed [13]. As recently demonstrated, for aqueous solutions, it would be possible to exploit the adsorptive capacity of polystyrene resin to remove the BTEX pollutants, (less than 1ppm) [14].

Biosensors are innovative tools to detect BTEX compounds in different environments for the pollution control. An example of them it is represented by the bio-reporter systems, based on the use of gene expression systems where the promoter activation by transcription regulator, in presence of a specific analyte, leads to the expression of the gene report making possible the detection and the quantization of the target analyte [15–17]. Whole-cell biosensor for BTEX detection was developed engineered genetically an *E*. *coli* strain expressing toluene dioxygenase (TDO) and toluene dihydrodiol dehydrogenase (TodD), enabling the conversion of BTEX into their respective catechols and then into colored products. The following detection limits were observed: a) benzene 10 μM; b) toluene10 μM; c) ethyl-benzene 20 μM; d) xylene 50 μM [18].

Analytical methods to detect benzene concentrations in air include absorption traps (Chromosorb 106, Tenax, Carbotrap and Carbosieve) [19] and subsequent separation by gas chromatography with detection by flame ionization, photo-ionization or mass chromatography [20]. Recently optical methods, such as differential optical absorption spectroscopy, have been developed for benzene detection [21, 22]. Other sensors (gas sensor) fabricated by a simple casting of single-walled carbon nanotubes (SWNTs) on an inter-digitized electrode (IDE), showed a linear response for concentrations of sub ppm to a few hundreds of ppm, with a detection limit of 20 ppm for benzene [23].

To date biosensors for specific detection of benzene levels in atmosphere are not yet reported in literature.

In this study we report on the identification of a protein belonging to lipocalin superfamily for applications related to benzene detection. Lipocalin proteins are secreted by mammal cells with different biological functions such recognizes and binds with different affinity to small hydrophobic and aromatic molecules [24, 25].

Odorant-binding proteins (OBPs) are members of lipocalins family. OBPs are able to transport odorant molecules from nasal mucosa to olfactory receptors. The transport is reversible, and it is possible to estimate the dissociation constant values that are on the order of micro-moles [26]. OBPs bind to a wide variety of ligands [26–31]. As previously reported, OBPs bind to 1-aminoanthracene (1-AMA), at micromolar concentration [31–35]. pOBP excited at 295 nm (where only the tryptophan residue absorbs) shows a fluorescence emission peak at 346 nm [36]. In the presence of 1-AMA the fluorescence emission of pOBP is quaenched and a new emission band centered at 481 nm is displayed. This phenomenon (fluorescence resonance energy transfer) is due to an energy transfer process between the pOBP tryptophan residues and the 1-AMA molecules present in the protein binding site [36]. Based on it, we developed a competitive fluorescence resonance energy transfer (FRET) assay to monitor benzene presence in atmospheric environment [32–34, 37–40].

Here, we performed a virtual screening analysis based on reverse docking and direct docking to select the best OBP to estimate the presence of atmospheric benzene. OBP from *sus scrofa* mucosa (pOBP) [27] resulted to be the best candidate as molecular recognition element (MRE) for a biosensor for benzene detection [28–31].

## Materials and methods

### Materials

The strain of *Escherichia coli* BL21 (DE3) with the genotype fhuA2 [lon] ompT gal (λ DE3) [dcm] ΔhsdS λ DE3 = λ sBamHIo ΔEcoRI-B int: (lacI: PlacUV5: T7 gene1) i21 Δnin5 was purchased from New England Biolabs (UK) Ltd. All solutions were prepared using deionized water, further purified using the Millipore Milli-Q system. The chemical standards, solvents and buffers were purchased from Sigma-Aldrich. The components used for protein electrophoresis were purchased from Bio-Rad. Other reagents were of analytical or higher grade available.

### Reverse docking: idTarget

The reverse docking methodology, by molecular docking simulations, allows for prediction of the binding affinity of a single molecule (ligand) versus a large number of protein structures (target). This methodology was used as screening to predict the benzene affinity of a specific family of proteins: the lipocalins. A reverse docking approach protocol was applied by using idTarget platform (http://idtarget.rcas.sinica.edu.tw/), a free web-server for the prediction of possible targets for the binding of small chemical molecules through a divide-and-conquer docking approach [41]. To ligand, idTarget automatically assigns the protonation state; concerning the scanning process, the ligand was mapped to the binding site of the homologous proteins structurally aligned and locally minimized by adaptive local searches to remove too close contacts with protein atoms [41]. The used search docking parameters were set to a default value. MEDock [42] was used to perform docking versus the set of lipocalins PDB entries, the docking poses was locally minimized and rescored by AutoDock4 [43] scoring function. Finally, the binding free energy value was calculated with the AutoDock4 scoring function. The three-dimensional structure of benzene (ligand) file in .sdf format was downloaded from PubChem (http://pubchem.ncbi.nlm.nih.gov/) [44]. Then this file was converted into .mol2 format using UCSF Chimera (http://www.rbvi.ucsf.edu/chimera/) [45] and submitted to idTarget server. The lipocalins (target) identified in RCSB PDB database (http://www.rcsb.org/) [46], using as key search word lipocalins, are 280 (update at December 31, 2015). Their pdb codes (**S1 Table**) were submitted to idTarget. The best targets in terms of predicted energy, inhibition constant and Z-score retrieved by idTarget were selected, downloaded from Protein Data Bank-PDB and analyzed. In addition, the resolution of the protein structure and the correctness of the structure were checked for the all selected targets. Finally, in order to consider for the next steps only strictly reliable results, those structures at resolution less than 2.0 Å or with missing residues and atoms were not considered.

### Direct docking: Autodock 4.2

The direct docking procedure, by molecular docking simulations with Autodock v.4.2 r. 4.2.6 [43], was used to evaluate the appropriate binding orientations and conformations of the ligand with the proteins identified by idTarget [41] screening. The docking procedure consists of several steps: a) preparation of the receptor and ligand files; b) calculation of the affinity maps through the 3D grid construction that envelops the ligand-receptor complex; c) definition of docking parameters and real simulation. The software used for the preparation and simulation stages are included in a single package: MGLTools (http://mgltools.scripps.edu/), which uses AutodockTools (ADT) 1.5.6 to prepare steps, Autogrid [47] to calculate the affinity maps and Autodock 4.2 to generate the poses that the ligand could assume and the binding free energy value calculation [43]. The 3D structure of the ligands was downloaded from PubChem [44] in .sdf format and converted into .pdb format with UCSF Chimera [45]. The structure files of selected lipocalins were downloaded in .pdb format by RCSB PDB [46]. The identification codes are 1DZK [48] and 1GT1 [49]. The integrity files were checked: the ligand heteroatoms of co-crystallization and the water molecules were eliminated. Only the chain A was considered for 1GT1. The ligand and receptor files were loaded in ADT. Through a series of intermediate steps all hydrogen atoms, chargers and atom types were added [50, 51]. The ligand torsdof was calculated, and the structures were protonated and a partial charge was assigned to atoms thus creating two different files with .pdbqt extension.

In addition, a grid box of 50×60×50 points, with a spacing of 0.375 Å, was used for all docking experiments. The grid encloses the receptor-ligand complex. By Autogrid, the affinity maps were calculated and the information obtained were saved in a .glg file format. 100 Docking runs were performed using the AutoDock Lamarckian genetic algorithm, treating the protein as rigid and the ligand as flexible. The number of evaluated poses to 30,000 and the maximum number of energy evaluations to 2,500,000 was set. All other parameters were set to default.

Docking poses were clustered using an RMSD value tolerance of 2.0Å. The docking simulation results, conducted by Autodock4.2, were collected in a file with .dlg extension containing the three-dimensional coordinates of the generated poses. The binding free energy and the histogram of the poses clusterization obtained, ordered in terms of geometrical difference, were compared to a reference co-crystallized ligand 1-aminoanthracene. The amino acid residues involved in the interactions with the ligand and the presence of H-bonding or π-π interactions were obtained by ADT. The residues involved in the interactions with the ligand were evaluated using as VWD (Van Der Waals radius) with a value of 1.15 Å. The best ligand-protein complexes were identified as those with the lowest binding energy and with the higher number of poses in the cluster. In order to check the parameters used during the docking simulations, a re-docking procedure was performed on the 1GT1structure and its co- crystallized ligand 1-AMA.

### Expression and purification of recombinant pOBP

A colony of *Escherichia coli* BL21 (DE3) cells transformed, by thermal-shock procedure, with the pGEX2TK expression vector containing the gene encoding the fusion protein (pOBP-GST) was picked and inoculated in 2.5 mL Luria-Bertani (LB) broth with ampicillin at the final concentration of 50 μg/mL, at 37°C, overnight, under shaking at speed of 160 rpm. Then, the culture was inoculated in 0.5 L of fresh LB medium containing ampicillin at the final concentration of 50 μg/mL, and was incubated at 37°C under shaking at speed of 160 rpm. When the culture absorbance value at 600 nm was equal to 1.0 O.D, the expression of the gene encoding recombinant protein was induced with isopropyl-β-D-1-thiogalactopyranoside (IPTG) at the final concentration of 0.5mM. After 3h of the post-induction incubation under shaking, the bacterial suspension was centrifuged at 3500 rpm for 30 minutes at 4°C. The cellular pellet obtained was re-suspended in (PBS) phosphate buffer saline (137mM NaCl, 2.7mM KCl, 10mM Na_2_HPO_4_, 1.8mM KH_2_PO_4_, pH 7.3), and was incubated, in the presence of 0.4% Lysozyme for 30 minutes at 37°C, followed by DNase I (50μg/ml per mL of solution) and 5mM MgCl_2_ (1 mg per ml of solution) at 37°C for 30 minutes. The cells were lysed using a French pressure cell (Aminco Co., Silver Spring, MD) at 2,000 psi (1 psi = 6.9 kPa). The nucleic acid fragments and cell debris were removed by centrifugation at 30000 rpm; the soluble fraction collected was concentrated with a 30,000 MWCO centrifugal filter device (Millipore), in order to execute also a first partial purification from high molecular weight contaminants (>30000 Da). Filtered sample was applied to a Glutathione Sepharose 4 Fast Flow resin (GE Healthcare, Life Sciences at Biocompare.com.) incubated at room temperature for 30 minutes under shaking. After incubation phase, several washes with the PBS were carried out to remove protein contaminants that are not bound to the resin. After the washing steps, the column was incubated with thrombin (1unit of thrombin per 100 μg of fusion protein) for 16 hours at 25°C, and then the pOBP was collected; at the end, with elution buffer (50mM Tris-HCl, 10mM reduced glutathione pH 8.0), GST-tag was removed from the resin. SDS-PAGE was carried out according to ref [52] to assess protein purity. The protein concentration was determined by the absorption spectra analysis between 220 and 320 nm. Spectra were recorded on a Jasco V730 UV/Vis spectrophotometer. The protein concentration was calculated on the basis of the absorbance values at 278 nm by using the Lambert and Beer’s law. The molar extinction coefficient is equal to 12200 M^-1^ cm^-1^ [49], the molecular weight is 17.71 KDa.

### Circular dichroism spectroscopy

Circular dichroism (CD) spectra were recorded using Jasco J-810 spectropolarimeter (Jasco, Tokyo, Japan) equipped with the Neslab RTE-110 temperature-controlled liquid system (Julabo F-25 MA Seelbach, Germany) and calibrated with a standard solution of (+)-l0-camphorsulfonic acid. Sealed cuvettes with a 0.1 cm and 1.0 cm path length (Helma, Jamaica, NJ) were used in the far-UV (200–240 nm) and near-UV (250–320 nm) region, respectively. Spectra were recorded at 2 nm intervals at a scan speed of 100 nm/min. Photomultiplier high voltage did not exceed 600 V in the spectral regions measured. The spectra were an average of 5 accumulated scans with subtraction of the baseline recorded for buffer solution and smoothed with Spectropolarimeter System Software (Jasco, Japan) version 1.00. Measurements were carried out under a nitrogen stream. The results are expressed in terms of molar ellipticity. Measurements was performed on homogeneous samples of 10μM pOBP in PBS buffer pH 7.4, at temperature increasing from 25°C to 85°C (far-UV) and in the presence of increasing concentrations of benzene (0.1–10 μM) (near-UV).

### Fluorescence spectroscopy

Steady-state fluorescence spectroscopy was used to evaluate the integrity of pOBP structure and its binding capacity. Fluorescence measurements were performed on FP 8600 Jasco fluorimeter equipped with a cell temperature-controlled sample holder. To selectively excite Trp residues of pOBP, the wavelength was set at 295 nm. Excitation and emission slit widths were fixed, at 2.5nm and 5nm, respectively. Emission spectra were recorded from 320 nm to 600 nm, at 1.0 nm intervals, at scan speed of 100nm/min using a 1.0 cm light path fluorimetric quartz cuvette. All measurements were carried out at 25°C with an accuracy of ±0.5°C, in PBS buffer pH 7.4, in the total volume of 500μl with 0.01% (v/v) ethanol final concentration [31]. The buffer alone was used as blank and its emission contribution was subtracted from the experimental spectra [53].

As extrinsic fluorophore was used 1-AMA dissolved in an ethanol solution. 1-AMA concentration was determined spectrophotometrically using an extinction coefficient equal to 35.45 mM^−1^cm^−1^ at 280nm. Protein sample concentrations were determined spectrophotometrically at 278 nm using extinction coefficient equal to 12200 M^−1^cm^−1^; to prevent the inner filter effect [54, 55] all measurements were carried out on a pOBP sample with an optical density value at 295nm lower than 0.1 OD, corresponding to 7μM protein concentration. Titration experiments were carried out with at increasing concentrations of 1-AMA from 0.05 to 10μM. The same setting was used for the competitive binding experiments with benzene; for chlorobenzene, biphenyl, penta-chlorobenzene and 2-acetylaminofluorene was used a pOBP sample at 10μM concentration. Taking into account the solubility coefficients of benzene in water, we supposed that it was possible to sample the atmosphere contaminants directly in a buffer solution. All measurements were carried out in triplicate, and the data obtained were analyzed with OriginLab 8.0 software.

### Virtual screening docking: PyRx

The methodology of the virtual screening allows to predict the binding affinity of several ligands versus a single protein structure (target). Virtual screening was used to predict the binding affinity of 156 selected air pollutants versus 1DZK. For the screening was used PyRx software [56], that uses Autogrid and Autodock4.2 algorithms to calculate the free energy of binding and develop the poses of interaction. The 1DZK target structure was downloaded in .pdb format by RCSB PDB [46], air pollutants list (178 entries at the 2015) was downloaded from US Environmental Protection Agency (EPA) web site at the section Toxics Release Inventory (TRI), subgroup Hazardous Air Pollutants (HAP’s) (https://iaspub.epa.gov/triexplorer/tri_text.list_chemical_hap) [57]. From this list were removed 40 molecules (metal compounds and other molecules because it was not possible to obtain 3D structures), then were added additional 18 known air pollutant molecules. The final list counted 156 molecules (**S2 Table**). The 1DZK target structure was loaded in PyRx in .pdb format and by a built-in function was prepared for analysis and converted in .pdbqt format. The identified air pollutants three-dimensional structures (**S2 Table**) downloaded from PubChem in .sdf format were loaded in PyRx, carried out an energy minimization by a built-in function based on MMFF94s force field [58] and finally, by a built-in function, were prepared as ligands for analysis in .pdbqt format. The parameters used for the grid were: 50x60x50 size, resolution 0.375Å. For the docking was used Lamarckian genetic algorithm with the generation of 100 poses for ligand, treating the protein as rigid and the ligand as flexible, and setting the number of evaluated poses to 30,000 and the maximum number of energy evaluations to 2,500,000. All other parameters were set to default. Docking poses were clustered using an RMSD value tolerance of 2.0Å. The docking simulation results, conducted by Autodock4.2, were collected in .dlg format files. The results were organized according to the lowest free energy of binding and with the higher number of poses in the cluster. Finally, the docking position of the ligand was evaluated for each ligand using the graphical interface.

## Results and discussions

### Identification of the molecular recognition element: An *in-silico* analysis

Since the lipocalin protein family is composed by many different proteins, to identify the molecular recognition element (MRE) able to bind with the higher affinity to benzene, it was used an *in-silico* experimental strategy. *In-silico* analysis, based on molecular docking simulations, was carried out in two complementary phases: an initial screening allowed narrowing of the investigation field and a second step of finishing identified lipocalin potentially with higher affinity to benzene. Reverse docking approach was used for the screening phase, using the IdTarget web-server; subsequently the results were subjected to direct docking experiments by Autodock 4.2.

During screening phase, by IdTarget web-server, were analysed 280 lipocalins identified in RCSB-PDB database. A single text file, including all lipocalins PDB codes **(S1 Table)**, and the benzene three-dimensional structure in .mol2 format, was submitted to IdTarget server.

**Fig 1:**
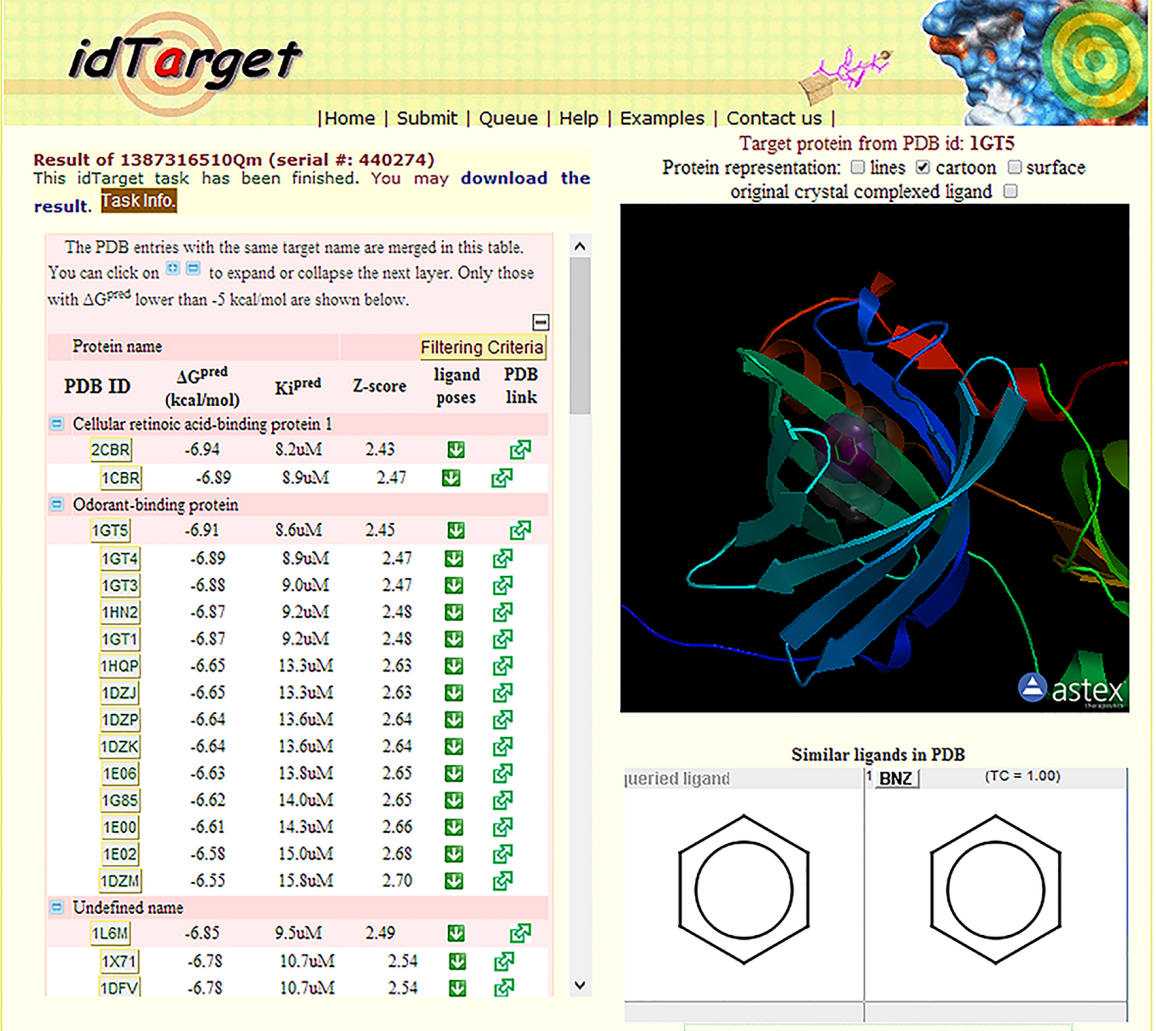
Reverse docking results. The graphical output provided by IdTarget.

IdTarget screening results, shown in **Fig 1,** are arranged in descending order on the basis of binding energy (ΔG^pred^), inhibition constant (Ki^pred^) and z-score value (z-score signifies an important target of the query ligand).

The most important optical features that we considered to select a protein target were:

1. Presence of only one tryptophan residue in the sequence, since it is necessary to preserve a stoichiometry with a ratio of 1:1 between the donor and acceptor molecules involved in the FRET phenomenon.
2. Accessibility of the tryptophan residue to the acceptor molecule (1-AMA).

Resulting analysis showed that 2CBR structure (cellular retinoic acid-binding protein 1) was the best target MRE, but this protein does not have suitable optical properties for utilizing it for the construction of a biosensor. IdTarget identified 14 OBPs with the correct features in order to use them for biosensors applications. All the identified OBP structures are relative to two OBP variants: the porcine OBP and the bovine OBP.

The relevant binding energy values obtained indicate a good interaction between the protein and ligand. The ΔG values are between -6.91 kcal/mol of bovine 1GT1 structure and the -6.55 kcal/mol of the structure 1DMZ porcine. IdTarget also provides graphic information on the benzene position in the OBP binding site, **Fig 1** shows that benzene is properly positioned into the beta barrel protein structure.

Two protein structures, 1GT1 (1.71 Å) as a representative of the bovine variant and 1DZK (1.48 Å) as a representative of the porcine variant, with the best crystallographic resolutions, were selected for the second stage of finishing by direct docking. The re-docking of 1GT1 structure (**S1 Fig**) allowed to set the parameters used for the simulation experiments with Autodock 4.2, prior to direct docking simulations.

**Fig 2:**
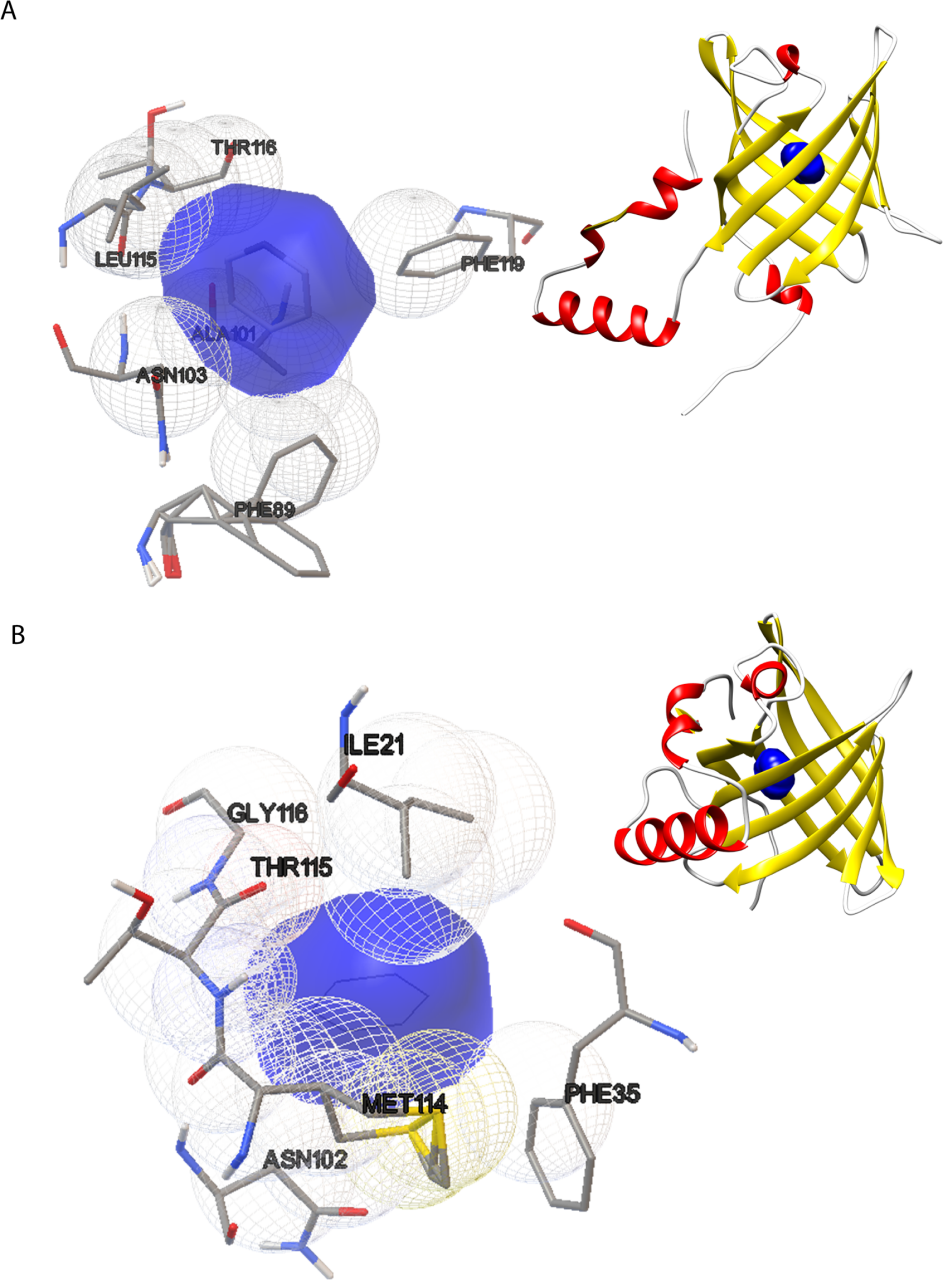
Direct docking simulation experiment results. (A) The drawing shows the conformation number 97, at the top right the position of benzene in the binding site of 1GT1 structure; at bottom (left) a particular binding site highlights the residues involved in the interaction with benzene. (B) The drawing shows the conformation number 91, at top (right) the position of benzene in the binding site of 1DZK structure; at bottom (left), a particular binding site highlights the residues involved in the interaction with benzene.

For the both 1GT1 and 1DZK structures were generated 100 conformations of the ligand-protein complex. They were analyzed according to three parameters: 1) predicted free energy of binding; 2) the most populous cluster and 3) the position of the ligand in the protein binding site. The protein conformation with lower predicted free energy of binding, belonging to the most populous cluster, was chosen among the obtained conformations; furthermore, it was evaluated the correct ligand positioning in the protein binding site, the amino acid residues involved in the interaction and the type of the predicted interactions. In **Table 1** and **Fig 2** are shown the direct docking simulation results. The interactions of the two protein structures with benzene are hydrophobic and Van der Walls types.

**Table 1:** Results of direct docking simulation experiments.

	**1DZK**	**1GT1**
**Representative conformation for the best cluster**	91	97
Constant of Inhibition (K_i_) mM	1,58	2,03
**Predicted binding energy (kcal/mol)**	-3,85	-3,67
**Number of poses in the best cluster**	68	56
**Amino acid residues involved in hydrophobic and van der Walls interactions**	ILE 21; PHE 35; ASN 102; MET 114; THR 115; GLY 116	PHE 89; ALA 101; ASN 103; LEU 115; THR 116; PHE 119

Results of direct docking simulations performed with Autodock 4.2.

Based on the results obtained from the docking simulation experiments, the 1DZK structure appears to have a higher affinity to benzene. However, small difference in binding energy values does not allow for the choice of one of the two structures on the basis of computational data; for this reason, the choice of the variant porcine protein (UniProt code P81245) as MRE, was also based on its structural features (e.g. the presence of a single tryptophan residue in position 16 in the binding site and because it is a monomeric protein).

### Purification of the porcine odorant-binding protein

The pOBP gene, identified by the number NM_213769.1, was a kind gift of Prof. Carlo Fini, University of Perugia. The recombinant plasmid encoding pOBP-GST was transformed into *E*. *coli* BL21(DE3). A colony derived from the *E*. *coli* strain transformed with the pGEX2TK expression vector, was grown in 10 mL of a LB medium with ampicillin at the concentration of 50μg/mL, overnight, then bacterial suspension was inoculated into 500 mL of fresh LB medium. When the culture reached the optical density value of 1.0, the expression of pOBP was induced by addition IPTG. After 3h at 37°C, the bacterial broth was centrifuged collecting 6 gr. pellet per liter of culture. The cellular pellet obtained was re-suspended in a lysis buffer and after treatment the purification of the protein was performed by GST-Affinity Chromatography using a Glutathione Sepharose 4 Fast Flow (GE Healthcare, Life Sciences at Biocompare.com.). The purity of the collected protein samples were analyzed by SDS-PAGE (data not shown). Fractions containing pOBP were also investigated by spectrophotometer to determine the protein concentration and to evaluate the presence of potential nucleic acid contaminants. The spectroscopic analysis revealed an Abs_280_/Abs_260_nm ratio of 1.62, according with the protein purity range (1.4–1.7). The final purification step yields a 6.0 mg/L protein solution.

Final concentration of pOBP, 1.15 mg/mL was determined by the Lambert and Beer’s law using molar extinction coefficient equal to 12200 M-1 cm-1.

### Circular dichroism measurements

To assess the thermal structural stability of the protein, circular dichroism (CD) measurements were performed in the far-UV and near-UV both in the absence and in the presence of increasing concentration of benzene.

To evaluate the protein thermal stability, CD measurements in the far-UV were carried out from 25°C to 85°C. The data shows that the CD spectrum of pOBP, collected in the far-UV at 25°C, is characterized by the presence of two absorption bands: a positive band at 200 nm and a negative band at 216 nm. These two bands may be related to the protein β-sheet content [59] of which is mainly composed pOBP structure [27].

**Fig 3:**
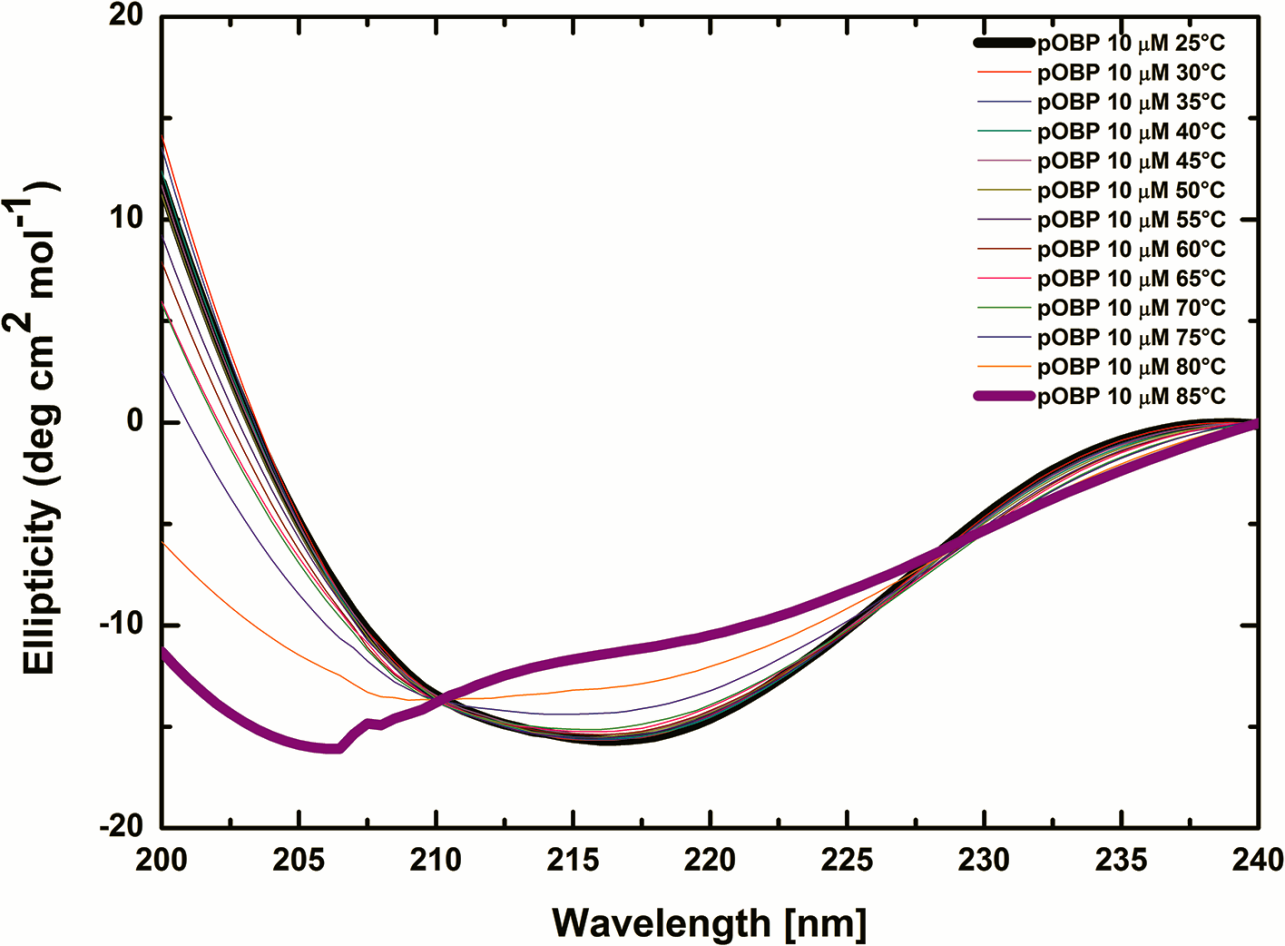
Thermal stability of pOBP secondary structure. Far-UV CD spectra of pOBP collected at increasing temperatures from 25°C to 85°C. It is possible to observe a variation of the two characteristic absorption bands at 200 nm and 216 nm and the presence of an *isodichroic* point at 211 nm.

CD measurements in far-UV, carried out at increasing temperature, show that the secondary structure content of pOBP changes from a predominantly beta-sheet structure content to a random coil content. Progressive reductions of absorption of the positive band at 200nm and the negative band at 216 nm, followed by a decrease of the absorption band at 222 nm [60], indicate the presence of a transition from ordered secondary structures to random structures (**Fig 3**). The intersection point at 211 nm (*isodichroic* point) of the different protein spectra, suggests the presence of an equilibrium state among protein β-sheet structures and unfolded structures. Finally, CD data also point out a high structural stability of the protein as already reported in [29].

To evaluate the effect of benzene on pOBP structure, far- and near-UV CD measurements were carried out at 25°C in the presence of increasing amounts of benzene.

**Fig 4:**
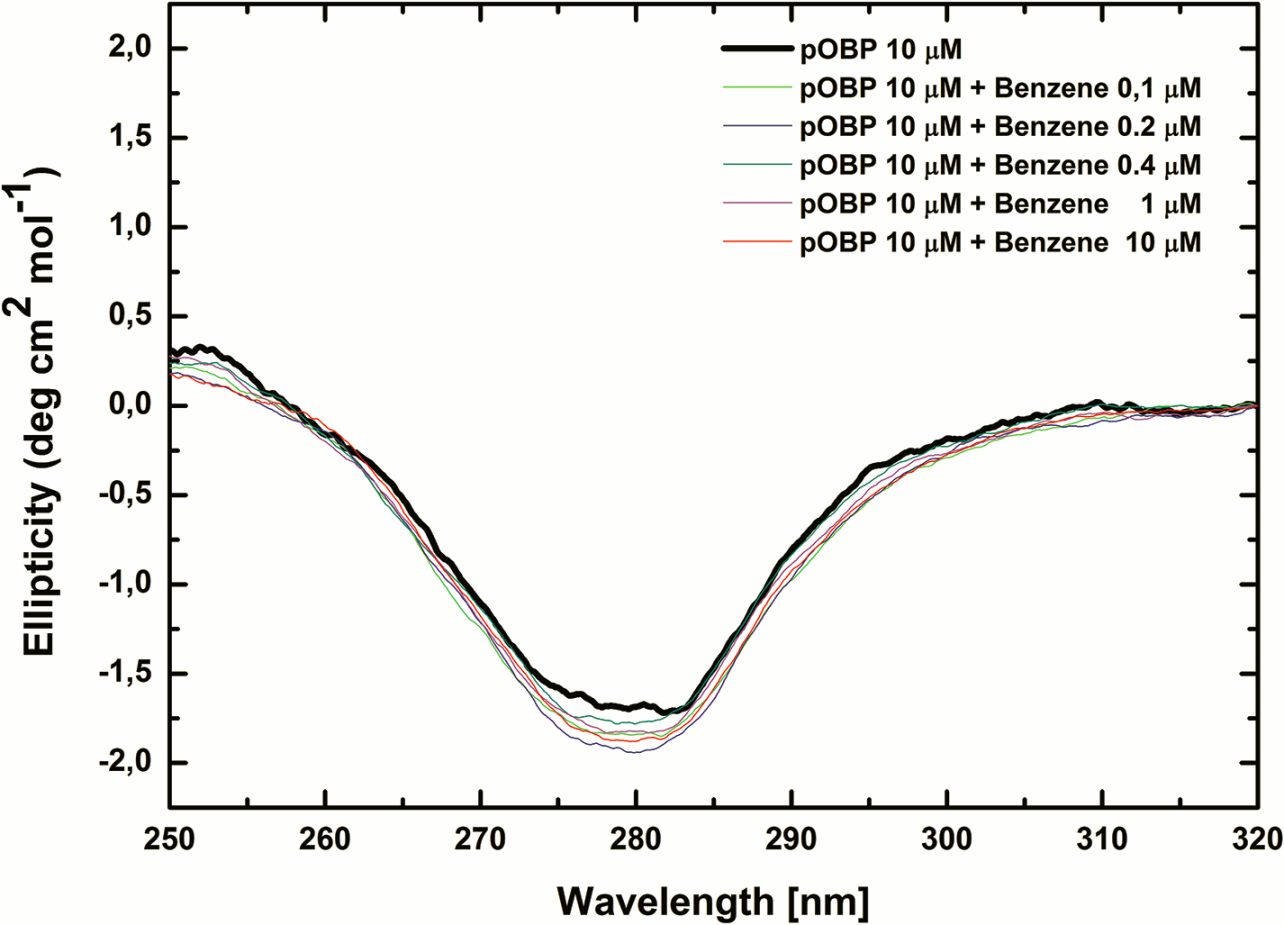
Benzene effect on pOBP structure. Near-UV CD spectra of pOBP collected at different concentrations of benzene (0–10 μM).

Far-UV data show no significant changes in pOBP secondary structure content (**S2 Fig**). On the contrary, near-UV data point out that the absorption band at 280 nm increases at increasing the concentration of benzene, suggesting an increasing flexibility of tryptophan and/or tyrosine residues side chains (**Fig 4**).

### Fluorescence spectroscopy measurements

**S3 Fig** shows the fluorescence emission spectra of pOBP upon excitation at 295 nm.

The emission spectrum of pOBP shows a maximum at 340 nm. The position of the fluorescence emission maximum is blue-shifted respect to the emission maximum of N-acetyl-tryptophanylamide (NATA) centered at 350 nm (data not shown), suggesting that the single tryptophan residue (W16) of pOBP is located in buried and/or un-relaxed protein microenvironments.

Fluorescent probes are widely used to study some properties of the proteins. Several extrinsic fluorescent probes have been used to assess the lipocalin interactions with ligands. The most used is the 1-aminoanthracene [31, 32].

**Fig 5:**
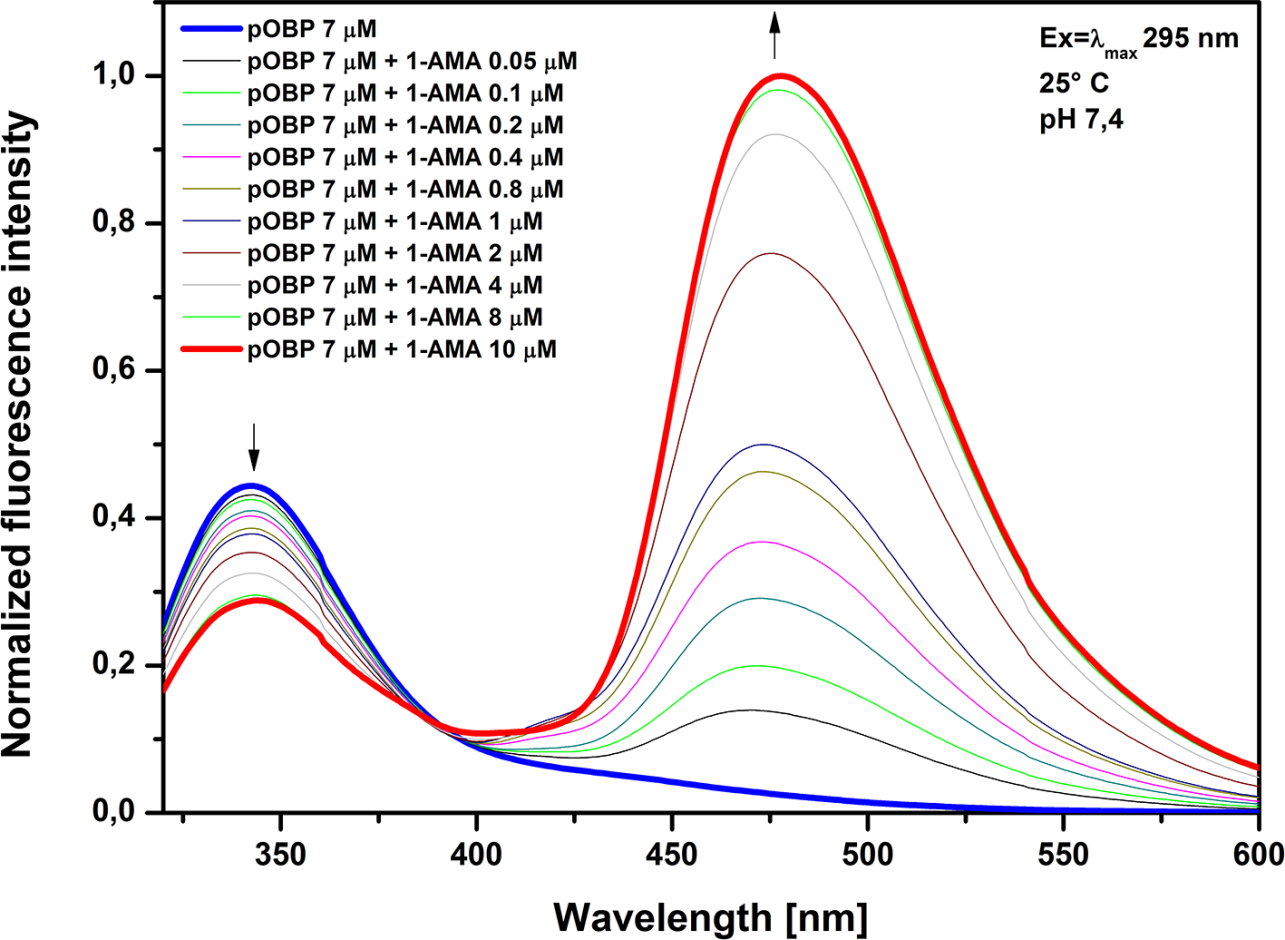
1-AMA titration curve spectra. Emission spectra of the pOBP excited at 295 nm in the absence and in the presence of different amounts of 1-AMA.

In the aqueous solution 1-AMA displays a weak fluorescence emission peak with a maximum at 537 nm, when excited at 295 nm. The addition of 1-AMA to pOBP solution induces a reduction of fluorescence intensity at 340 nm and an increase of fluorescence intensity at 481 nm (**Fig 5**). This fluorescence change may be explained by the phenomenon of fluorescence resonance energy transfer (FRET) process between the protein tryptophan at 16 position (acting as a donor) and 1-AMA (acting as acceptor) intercalated in the binding site of the protein.

**Fig 6:**
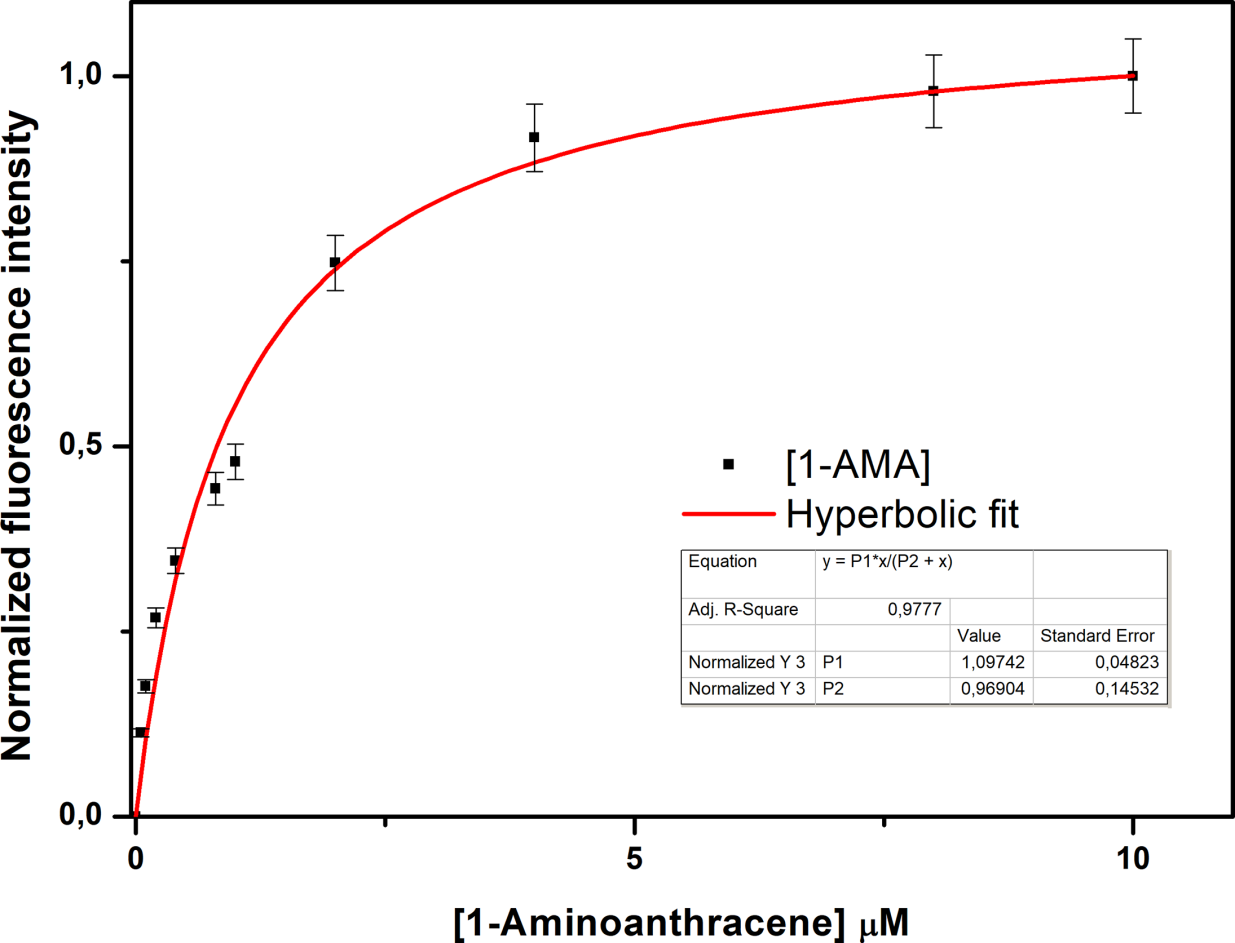
1-AMA titration curve fitting. Plot of the intensity increase of 1-AMA fluorescence intensity as a function of fluorophore concentrations.

The presence of an isosbestic point at 388 nm indicates the presence of an equilibrium between the protein alone and the protein in complex with 1-AMA, suggesting the absence of an intermediary of reaction and confirming that a binding phenomenon occurs (**Fig 5**). Furthermore, the analysis of the binding curve of the 1-AMA to pOBP (**Fig 6**), allows to identify a plateau point value, which indicates the saturation of the binding sites of protein by the concentration of 1-AMA.

### Competitive FRET assay for benzene binding to pOBP

**Fig 7:**
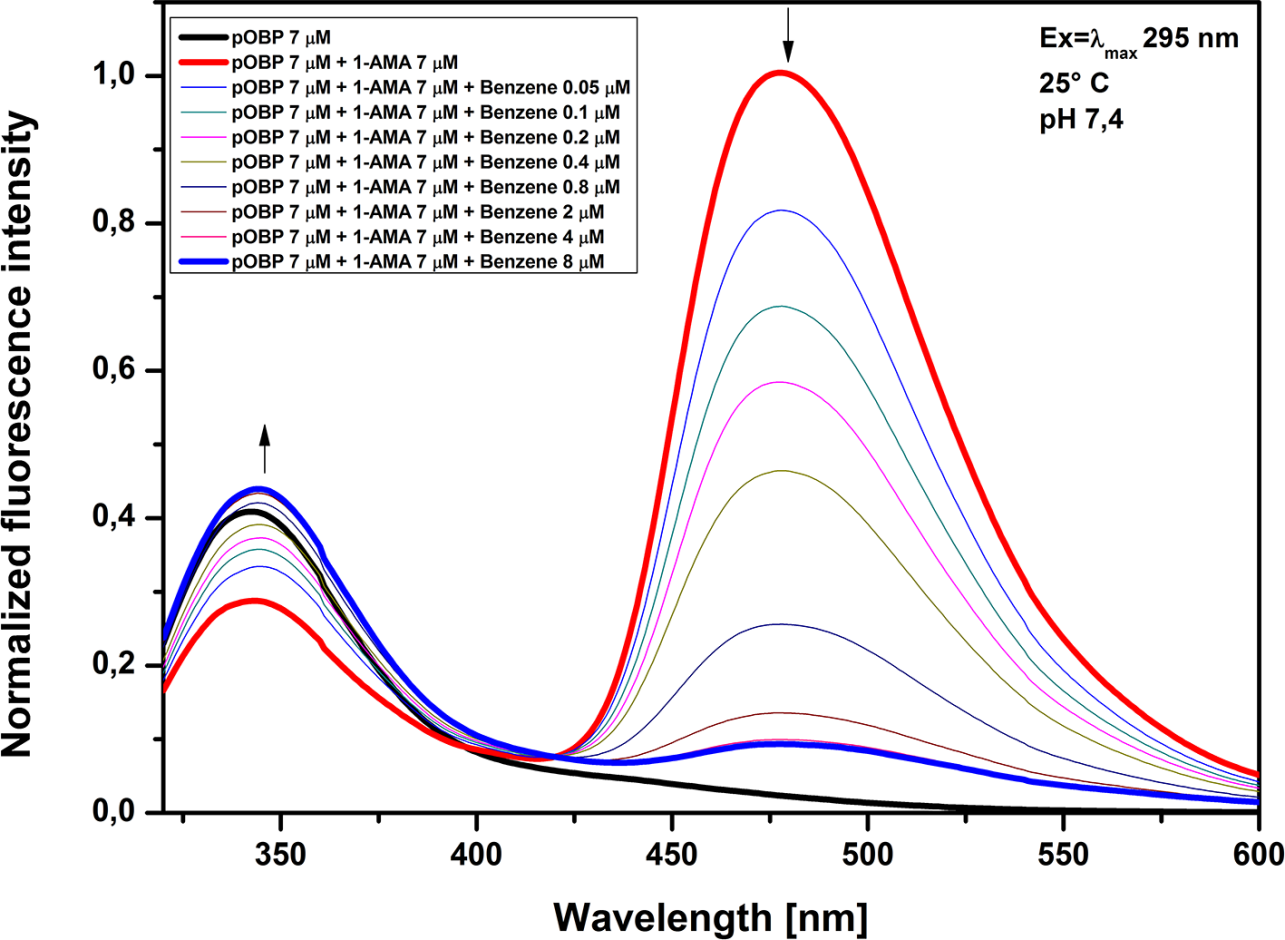
Fluorescence emission spectra at increasing concentrations of benzene. Figure shows the emission fluorescence spectra of pOBP in the presence of saturating concentrations of 1-AMA. The addition of increasing amounts of benzene determines a decrease of the peak at 481 nm and an increase of the peak at 340 nm.

A competitive FRET assay to determine benzene binding to pOBP was developed using 1-AMA. This assay was designed from docking simulation results that suggest that pOBP binds to benzene. Docking simulation experiments showed both 1-AMA position within the binding site of pOBP and the protein amino acid residues involved in the interaction with the 1-AMA (**S4A Fig**). A partially superimposition of the obtained 1-AMA and benzene docking pose in the protein binding site shows (**S4B Fig**) that benzene and 1-AMA fill the same region of the binding site; consequently, most of the amino acid residues involved in the interaction with benzene and 1-AMA appear to be in common. For this reason, benzene could compete with 1-AMA to binding site of pOBP. A competitive assay was designed and binding of benzene to the binding site of pOBP was evaluated by monitoring the displacement of 1-AMA at increasing concentrations of benzene (**Fig 7**).

**Fig 8:**
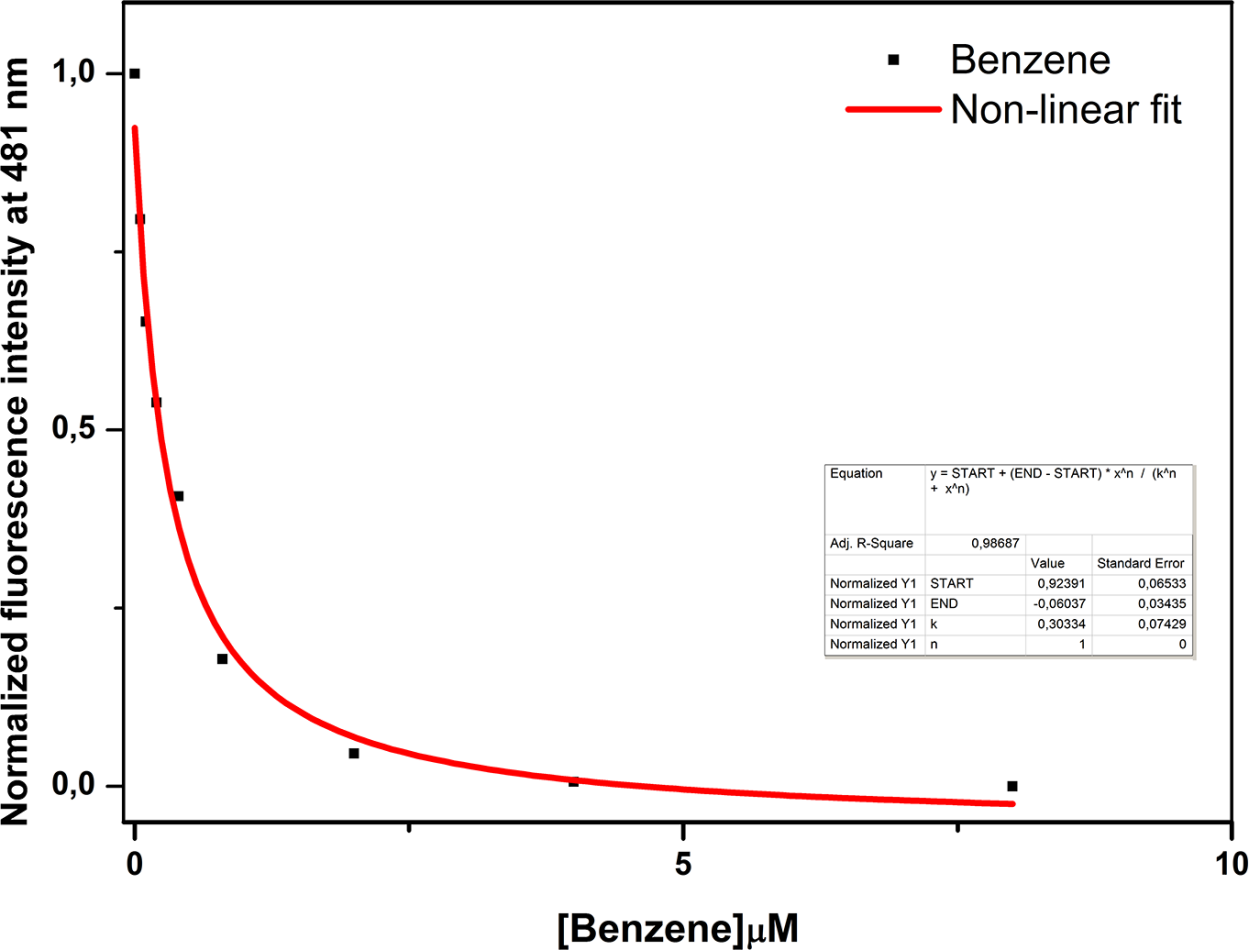
Benzene titration curve fitting. Plot of the intensity decrease of 1-AMA fluorescence emission at 481 nm as a function of benzene concentration. In red color it is shown the fitting curve obtained by a non-linear function.

In **Fig 8** is shown the titration curve of benzene interaction to pOBP. The decrease of the fluorescence intensity at 481 nm as a function of increasing concentrations of benzene allows us to calculate the kinetics parameters of the interaction. The dissociation constant value of 0.30 ± 0.07 μM was calculated by using a non-linear fitting function. The software used was OriginPro8 (OriginLab Corporation, Northampton, MA, USA). The minimum detectable benzene concentration is 0.05 μM, equivalent to 3.9μg/m^3^. The emission limit value in the atmosphere for benzene, established by Directive 2008/50/EC is 5μg/m^3^, and then the developed assay can be useful [61].

### pOBP binding specificity to benzene

To evaluate the specificity of benzene binding to pOBP, we identified and tested putative interfering air pollutants. By virtual screening docking simulation, it was predicted the affinity of the 156 identified air pollutants to pOBP (**S2 Table)**.

Results of the virtual screening were re-organized to reduce the number of molecules to be experimentally tested; the discrimination criteria used were: 1) accessibility of the molecules or the binding site of the protein; 2) binding affinity assessed in terms of the predicted binding energy; 3) molecule solubility in water; 4) chemical features of the molecules.

The accessibility to binding site of the protein was evaluated for each molecule tested by PyRx graphic viewer. From a total of 156 molecules tested, only 87 of them have access to the protein binding site. For these 87, we identified the pose with a lower binding energy belonging to the most populous cluster. These 87 molecules (ordered based on the binding energy predicted by virtual screening and using as a threshold value the binding energy value of the pOBP-benzene complex (-3.85 Kcal/mol)) were classified into two groups: a) 55 molecules were identified as molecules with a lower binding energy; b) 32 molecules were identified as molecules with binding energy greater than -3.82 kcal/mol. Since the competitive assay was carried out in aqueous buffer, 7 molecules insoluble in water (reported in **Table 2)** were excluded from the analysis. The rest of 48 molecules were classified on the basis of their chemical nature: 1) aromatic; 2) aliphatic; 3) halogenated or no-halogenated; 4) nitrogenous or no-nitrogenous; 5) ordered (**Table 2**) in according to the lowest binding energy.

**Table 2:** Air pollutant molecules that bind to pOBP.

NAME	PUBCHEM ID	ΔG (Kcal/mol)	SOLUBILITY^(a)^ (mg/L)	NATURE^(b)^
HEPTACHLOR	3589	-8,07	I	
TOXAPHENE	5284469	-7,73	3,0	RC
CAPTAN	8606	-7,65	5,1	RNS
4,4'-METHYLENEBIS(2-CHLOROANILINE)	7543	-7,00	I	
2-ACETYLAMINOFLUORENE	5897	-6,80	6,3	AN
BIPHENYL	7095	-6,55	7,5	A
3,3'-DICHLOROBENZIDINE	7070	-6,53	700	CAN
3,3'-DIMETHYLBENZIDINE	8413	-6,50	1300	AN
DIBUTYL PHTHALATE	3026	-6,48	11	A
DIBENZOFURAN	568	-6,33	3,1	A
PENTACHLOROBENZENE	11855	-6,15	0,8	AC
1,2-DIPHENYLHYDRAZINE	31222	-6,06	221	AN
4,4'-METHYLENEDIANILINE	7577	-6,06	I	
HEXACHLOROBENZENE	8370	-5,97	I	
CARBARYL	6129	-5,89	110	AN
4-AMINOBIPHENYL	7102	-5,84	I	
HEXACHLOROCYCLOPENTADIENE	6478	-5,78	1,8	AC
PENTACHLOROPHENOL	992	-5,77	14	AC
BENZOIC TRICHLORIDE	7367	-5,55	I	
BENZIDINE	7111	-5,53	322	AN
1,2,4-TRICHLOROBENZENE	13	-5,43	49	AC
NAPHTHALENE	931	-5,40	31	A
PHTHALIC ANHYDRIDE	6811	-5,29	6000	A
2,4,6-TRICHLOROPHENOL	6914	-5,28	500	AC
2,4,5-TRICHLOROPHENOL	7271	-5,17	1200	AC
PARATHION	991	-5,13	11	A
QUINOLINE	7047	-5,12	6110	AN
PROPOXUR	4944	-5,07	1860	AN
DIMETHYL PHTHALATE	8554	-5,06	4000	A
QUINTOZENE	6720	-5,00	0,4	CAN
CUMENE	7406	-4,99	61	A
TOLUENE-2,4-DIISOCYANATE	11443	-4,77	I	
1,4-DICHLOROBENZENE	4685	-4,76	79	AC
ACETOPHENONE	7410	-4,64	6130	A
HEXACHLOROETHANE	6214	-4,61	50	RC
M-XYLENE	7929	-4,54	160	A
O-XYLENE	7237	-4,47	180	A
STYRENE	7501	-4,45	300	A
ETHYLBENZENE	7500	-4,40	170	A
4-(2,4-DICHLOROPHENOXY)BUTANOIC ACID	1489	-4,39	46	AC
P-CRESOL	2879	-4,38	21500	A
BENZYL CHLORIDE	7503	-4,36	525	AC
O-TOLUIDINE	7242	-4,34	16600	AN
N,N-DIMETHYLANILINE	949	-4,31	1454	AN
M-CRESOL	342	-4,28	22200	A
CHLOROBENZENE	7964	-4,25	499	AC
TETRACHLOROETHYLENE	31373	-4,20	15	RC
STYRENE OXIDE	7276	-4,19	3000	A
O-CRESOL	335	-4,18	25900	A
2,4-DIAMINOTOLUENE	7261	-4,17	31800	AN
TOLUENE	1140	-4,07	526	A
O-ANISIDINE	7000	-4,00	13000	AN
HYDROQUINONE	785	-3,92	72000	A
ANILINE	6115	-3,90	36000	AN
BENZENE	241	-3,85	1770	A

Table reports, ordered in according to the lowest binding energy, the 48 molecules that have access to pOBP binding site with a predicted binding energy lower than pOBP-benzene complex; the solubility in water and chemical nature are also reported.

^(a)^HSDB database https://toxnet.nlm.nih.gov/newtoxnet/hsdb.htm.

^(b)^A = aromatic; R = aliphatic; C = halogenate; N = nitrogenous.

I = insoluble in water.

To identify the common features of the molecules that drive their binding to pOBP, the molecules were combined with each other, and divided into eight subclasses: 1) aromatic/halogenated; 2) aromatic/no-halogenated; 3) aromatic/nitrogenous; 4) aromatic/no-nitrogenous; 5) aliphatic/halogenated; 6) aliphatic/no-halogenated; 7) aliphatic/nitrogenous; 8) aliphatic/no-nitrogenous. From this analysis, the aromaticity is resulted as a predominant feature. Indeed only 4 molecules were aliphatic and therefore no longer taken into account in the subsequent step. Combining the halogenated or not-halogenated and nitrogenous or not-nitrogenous features, the group of aromatic molecules was divided in four subclasses and ordered based on the predicted binding energy, as reported in **Table 3**. The three most populous subclasses are: 1) aromatic not-halogenated and not-nitrogenous including 20 molecules; 2) aromatic nitrogenous including 12 molecules; 3) aromatic halogenated including 10 molecules. From each of these three groups, it was selected, and then experimentally tested by a competitive assay, the following molecules with the lowest binding energy value: 1) biphenyl; 2) penta-chlorobenzene; 3) 2-acetylaminofluorene; 4) chlorobenzene.

**Table 3:** Aromatic molecules subclasses.

NAME	ΔG (Kcal/mol)	NATURE^(a)^
***BIPHENYL***	***-6*.*55***	***A***
DIBUTYL PHTHALATE	-6.48	A
DIBENZOFURAN	-6.33	A
NAPHTHALENE	-5.40	A
PHTHALIC ANHYDRIDE	-5.29	A
PARATHION	-5.13	A
DIMETHYL PHTHALATE	-5.06	A
CUMENE	-4.99	A
ACETOPHENONE	-4.64	A
M-XYLENE	-4.54	A
O-XYLENE	-4.47	A
STYRENE	-4.45	A
ETHYLBENZENE	-4.40	A
P-CRESOL	-4.38	A
M-CRESOL	-4.28	A
STYRENE OXIDE	-4.19	A
O-CRESOL	-4.18	A
TOLUENE	-4.07	A
HYDROQUINONE	-3.92	A
BENZENE	-3.85	A
***PENTACHLOROBENZENE***	***-6*.*15***	***AC***
HEXACHLOROCYCLOPENTADIENE	-5.78	AC
PENTACHLOROPHENOL	-5.77	AC
1,2,4-TRICHLOROBENZENE	-5.43	AC
2,4,6-TRICHLOROPHENOL	-5.28	AC
2,4,5-TRICHLOROPHENOL	-5.17	AC
1,4-DICHLOROBENZENE	-4.76	AC
4-(2,4-DICHLOROPHENOXY)BUTANOIC ACID	-4.39	AC
BENZYL CHLORIDE	-4.36	AC
***CHLOROBENZENE***	***-4*.*25***	***AC***
3,3'-DICHLOROBENZIDINE	-6.53	ACN
QUINTOZENE	-5.00	ACN
***2-ACETYLAMINOFLUORENE***	***-6*.*80***	***AN***
3,3'-DIMETHYLBENZIDINE	-6.50	AN
1,2-DIPHENYLHYDRAZINE	-6.06	AN
CARBARYL	-5.89	AN
BENZIDINE	-5.53	AN
QUINOLINE	-5.12	AN
PROPOXUR	-5.07	AN
O-TOLUIDINE	-4.34	AN
N,N-DIMETHYLANILINE	-4.31	AN
2,4-DIAMINOTOLUENE	-4.17	AN
O-ANISIDINE	-4.00	AN
ANILINE	-3.90	AN

Table reports the 44 aromatic molecules organized in four subclasses: aromatic only, aromatic halogenated, aromatic halogenated nitrogenous and aromatic nitrogenous. Molecules in each subclass were ordered based on the binding energy value. In bold italic were highlighted the molecules selected to experimentally test.

^(a)^A = aromatic; C = halogenate; N = nitrogenous.

The last molecule was selected to clarify if the number of halogenation may affect the binding specificity of the protein.

### Competitive assay versus interfering pollutant molecules

The pOBP binding specificity was experimentally evaluated for four of the selected molecules by FRET competitive assay. The 2-acetylaminofluorene was not tested since its insolubility in aqueous buffer (6.3 mg/L).

**Fig 9:**
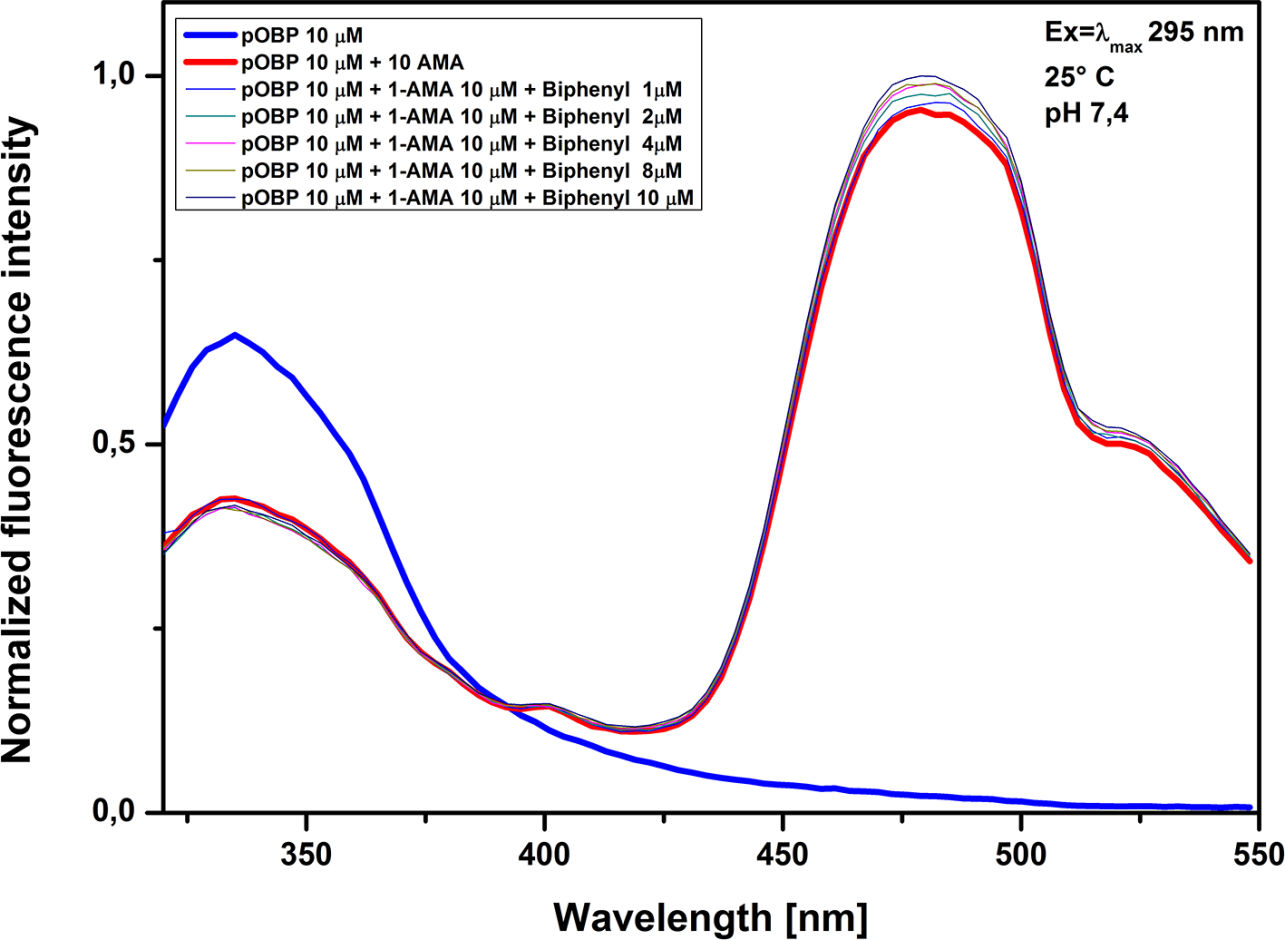
Biphenyl competitive assay. Figure shows the competitive assay. The competitive molecule used was biphenyl; fluorescence signal at 481 nm was unaffected.

**Fig 10:**
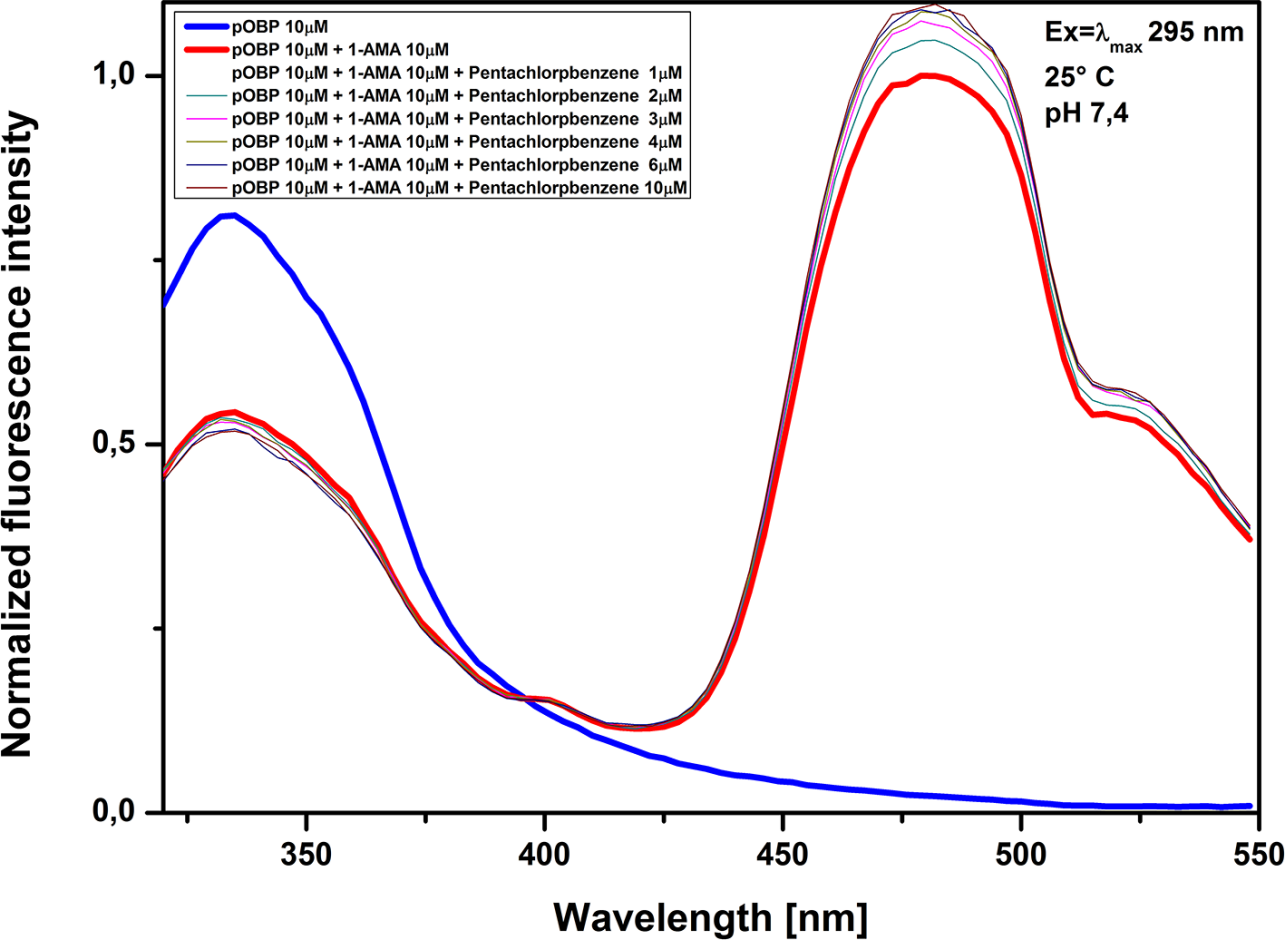
Penta-chlorobenzene competitive assay. Figure shows the competitive assay. The competitive molecule used was penta-chlorobenzene; fluorescence signal at 481 nm was unaffected.

**Fig 11:**
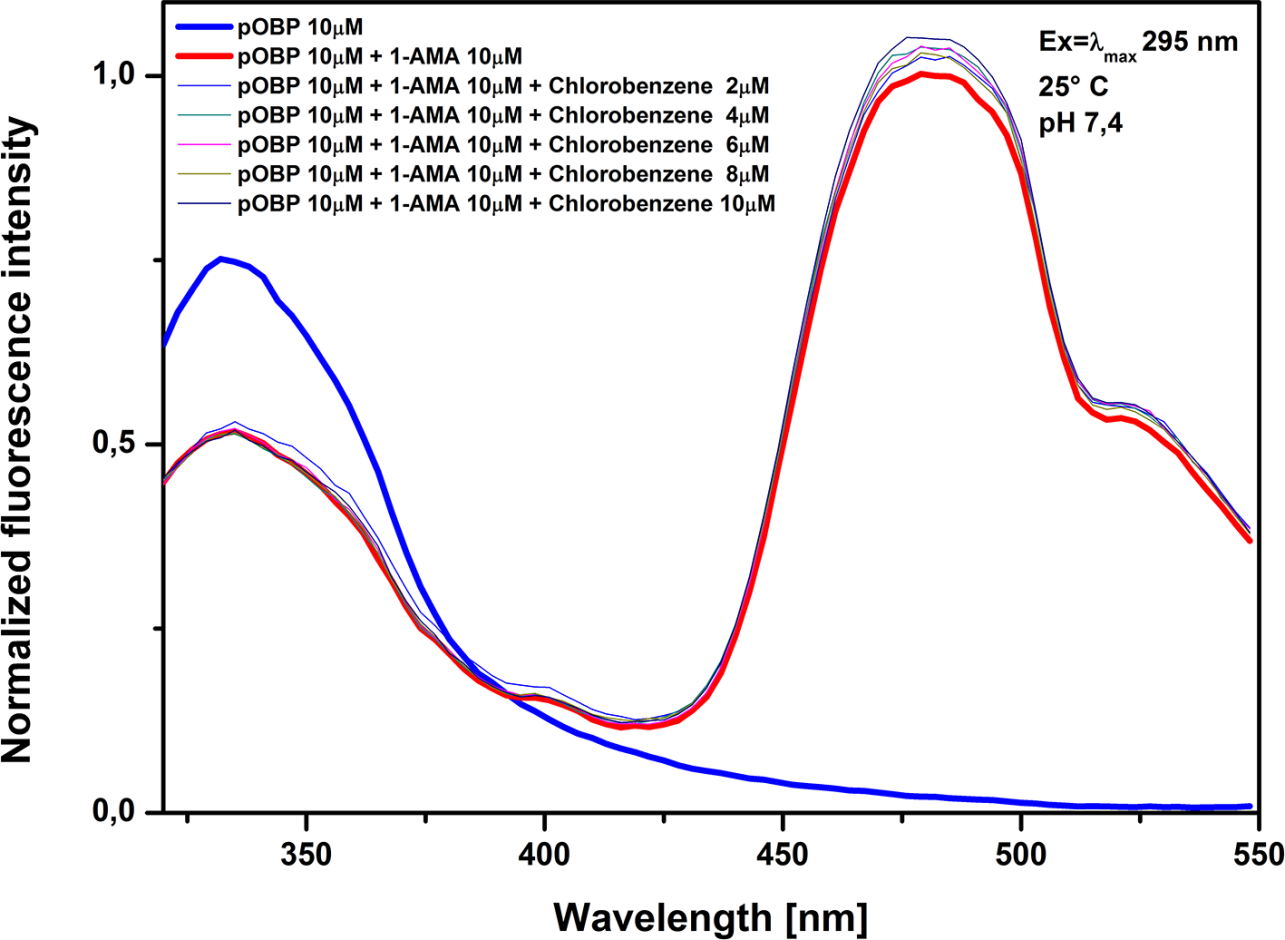
Chlorobenzene competitive assay. Figure shows the competitive assay. The competitive molecule used was chlorobenzene; fluorescence signal at 481 nm was unaffected.

The assays were carried out with a pOBP solution saturated of 1-AMA and tested at increasing concentrations of biphenyl (**Fig 9**), penta-chlorobenzene (**Fig 10**) and chlorobenzene (**Fig 11**). The increasing of pollutant concentrations did not effect the optical signal; no reduction in the fluorescence intensity at 481 nm was observed, suggesting that the 1-AMA is not displaced from the binding site of pOBP. On the other hand, a slight increase of the fluorescence intensity at 481 nm was observed, that could be considered not relevant if it is compared with the reduction of the fluorescence signal that occurs in the presence of benzene. Based on these results it is conceivable a good binding specificity of pOBP to benzene. However, it is noteworthy that the number of potential contaminants experimentally tested is not enough to allow to completely exclude that other molecules could interfere with the assay.

## Conclusion

*In-silico* analysis, by reverse and direct molecular docking approach, allowed us to identify pOBP as protein that binds benzene with high affinity and specificity. Purified pOBP was characterized by biochemical and spectroscopic methodologies, to evaluate the structural behavior to temperature and to the presence of benzene.

The pOBP showed high thermal stability in solution. In fact, the circular dichroism measurements proved that the pOBP preserves a structural stability up to 65°C.

Molecular recognition properties and structural peculiarities of pOBP were used to develop a competitive FRET assay for benzene determination. The developed assay displayed a high affinity for benzene detection with a LOD value of 0.05 μM (3.9μg/m^3^). This value is compatible with the European standard limit values for benzene in atmosphere. The binding specificity of pOBP to benzene was evaluated by *in-silico* and *in-vitro* approaches. Virtual screening experiments were performed, and the pOBP binding affinity constants of 156 atmospheric pollutant molecules were studied. Even if it was not possible to exclude that different molecules could bind to pOBP, our assay could be used as first-line detector to sense the presence of benzene in atmosphere. In fact, due to the high protein stability and the well-known purification method to yield large amounts of pOBP, it is possible to envisage the design of a robust optical biosensor to continuously monitor the level of benzene in atmosphere.

## Supporting information

S1 Table Table reports PBD codes of 280 lipocalin structures deposited in the RCSB-PDB database. (DOCX)

S2 Table Table reports names of 156 air pollutant molecules, with respective PUBCHEM ID, screened by PyRx. (DOCX)

S1 Fig Re-docking experiment of 1GT1in complex with 1-AMA, in yellow the co-crystallized ligand and in red the re-docking ligand. (TIF)

S2 Fig Far-UV CD spectra of pOBP collected in the presence of increasing concentrations of benzene (0–10 μM) at 25°C. (TIF)

S3 Fig Fluorescence emission spectra of pOBP tryptophan residue excited at 295 nm. (TIF)

S4 Fig (A) On the left, a detail of pOBP binding site with highlighted amino acid residues involved in the interaction with the 1-AMA; on the right, the position of 1-AMA in the binding site of pOBP. (B) On the left, a superposition of benzene and 1-AMA docking pose into the binding site of pOBP, on the right, a particular where it is possible to appreciate the two molecules to share the protein binding site region.
(TIF)

## References

[1] Lim SS, Vos T, Flaxman AD, Danaei G, Shibuya K, Adair-Rohani H, et al A comparative risk assessment of burden of disease and injury attributable to 67 risk factors and risk factor clusters in 21 regions, 1990–2010: a systematic analysis for the Global Burden of Disease Study 2010. Lancet. 2012;380(9859):2224–60. Epub 2012/12/19. 10.1016/S0140-6736(12)61766-8 ; PubMed Central PMCID: PMC4156511. 23245609 PMC4156511

[2] WHO. Burden of disease from Ambient Air Pollution for 2012—Summary of results, World Health Organization 2014;2014a. Available from: http://www.who.int/phe/health_topics/outdoorair/databases/AAP_BoD_results_March2014.pdf. Accessed 18 August 2014.

[3] WHO. Effects of air pollution on children's health and development: a review of the evidence, World Health Organization, Regional Office for Europe, Copenhagen 2005;2005a. Available from: http://www.euro.who.int/_data/assets/pdf_file/0010/74728/E86575.pdf.

[4] WHO. Review of evidence on health aspects of air pollution—REVIHAAP Project, Technical Report, World Health Organization, Regional Office for Europe, Copenhagen 2013;2013a. Available from: http://www.euro.who.int/__data/assets/pdf_file/0004/193108/REVIHAAP-Final-technical-report-final-version.pdf?ua=1. 27195369

[5] Lide DR. CRC Handbook of Chemistry and Physics. 89th ed. In: CRC Press/Taylor and Francis, Boca Raton, FL 2008:pp. 3–32.

[6] EU. Directive 2009/30/EC of the European Parliament and of the Council of 23 April 2009 amending Directive 98/70/EC as regards the specification of petrol, diesel and gas-oil and introducing a mechanism to monitor and reduce greenhouse gas emissions and amending Council Directive 1999/32/EC as regards the specification of fuel used by inland waterway vessels and repealing Directive 93/12/EEC (OJ L 140, 5.6.2009, pp. 88–113) 2009;2009a. Available from: http://eur-lex.europa.eu/LexUriServ/LexUriServ.do?uri=OJ:L:2009:140:0088:0113:EN:PDF.

[7] EEA. Air quality in Europe—2016 report, EEA Report No 28/2016. European Environment Agency. 2016:Available from: http://www.eea.europa.eu/publications/air-quality-in-europe-2016.

[8] David LB, Chao W, Jarabek A, Babasaheb S, Valcovic L. U.S. EPA. Carcinogenic Effects of Benzene: An Update (Draft Report). US Environmental Protection Agency, Office of Research and Development, National Center for Environmental Assessment, Washington Office, Washington, DC 1998;EPA/600/P-97/001F.

[9] IARC. IARC Monographs on the evaluation of carcinogenic risks to humans, 100F A review of human carcinogens. Part F: Chemical agents and related occupations. IARC Working Group on the Evaluation of Carcinogenic Risks to Humans: Lyon, France 2009;Available from: http://monographs.iarc.fr/ENG/Monographs/vol100F/mono100F.pdf. PMC4781612 23189753

[10] EU. Directive 2008/50/EC of the European Parliament and of the Council of 21 May 2008 on ambient air quality and cleaner air for Europe. 2008;OJ L 152:1–44. Available from: http://eur-lex.europa.eu/LexUriServ/LexUriServ.do?uri=OJ:L:2008:152:0001:0044:EN:PDF.

[11] Mukherjee AK, Chattopadhyay BP, Roy SK, Das S, Mazumdar D, Roy M, et al Work-exposure to PM10 and aromatic volatile organic compounds, excretion of urinary biomarkers and effect on the pulmonary function and heme-metabolism: A study of petrol pump workers and traffic police personnel in Kolkata City, India. J Environ Sci Health A Tox Hazard Subst Environ Eng. 2016;51(2):135–49. Epub 2015/11/21. 10.1080/10934529.2015.1087740 . 26587917

[12] Prati MV, Costagliola MA, Quaranta F, Murena F. Assessment of ambient air quality in the port of Naples. J Air Waste Manag Assoc. 2015;65(8):970–9. Epub 2015/06/02. 10.1080/10962247.2015.1050129 . 26029862

[13] Cruz L, Alve L, Santos A, Esteves M, Gomes Í, Nunes L. Assessment of BTEX Concentrations in Air Ambient of Gas Stations Using Passive Sampling and the Health Risks for Workers. Journal of Environmental Protection. 2017;8 (1):12–25. 10.4236/jep.2017.81002

[14] Thobeka P, Sudesh R. Investigation of BTEX compounds adsorption onto polystyrenic resin. South African Journal of Chemical Engineering. 2017;23:71–80. 10.1016/j.sajce.2017.03.001

[15] Hernandez-Sanchez V, Molina L, Ramos JL, Segura A. New family of biosensors for monitoring BTX in aquatic and edaphic environments. Microb Biotechnol. 2016;9(6):858–67. Epub 2016/10/21. 10.1111/1751-7915.12394 ; PubMed Central PMCID: PMC5072201. 27484951 PMC5072201

[16] Yu Q, Li Y, Ma A, Liu W, Wang H, Zhuang G. An efficient design strategy for a whole-cell biosensor based on engineered ribosome binding sequences. Anal Bioanal Chem. 2011;401(9):2891–8. Epub 2011/09/29. 10.1007/s00216-011-5411-7 . 21947012

[17] Burlage RS, Kuo CT. Living biosensors for the management and manipulation of microbial consortia. Annu Rev Microbiol. 1994;48:291–309. Epub 1994/01/01. 10.1146/annurev.mi.48.100194.001451 . 7826008

[18] Xu Zhaohui, Mulchandani Ashok, Chen W. Detection of Benzene, Toluene, Ethyl Benzene, and Xylenes (BTEX) Using Toluene Dioxygenase-Peroxidase Coupling Reactions Biotechnology Progress. 2003;19(6):1812–5. 10.1021/bp0341794 14656160

[19] Brown RHJ, Charlton J., Saunders K. J. The development of an improved diffusive sampler. Am Ind Hyg Assoc J. 1981;42(12):865–9. Epub 04 Jun 2010. 10.1080/15298668191420828

[20] Camagni SS P., editor. Optical Remote Sensing of Air Pollution. 1st Edition ed: Elsevier Science; 1984.

[21] Barrefors G. Monitoring of benzene, toluene and p-xylene in urban air with differential optical absorption spectroscopy technique. Science of the total environment. 1996;189–190:287–92. 10.1016/0048-9697(96)05221-7

[22] WHO. Air Quality Guidelines - 2rd ed Chapter 5.2 Benzene. Regional Office for Europe: Copenhagen Denmark 2000.

[23] Li J, LY, Ye Q, Cinke M, Han J and Meyyappan M. Carbon Nanotube Sensors for Gas and Organic Vapor Detection. NANO LETTERS. 2003;3(7):929–33. Epub 06/13/2003. 10.1021/nl034220x

[24] Flower DR. Multiple molecular recognition properties of the lipocalin protein family. J Mol Recognit. 1995;8(3):185–95. Epub 1995/05/01. 10.1002/jmr.300080304 . 8573354

[25] Flower DR. The lipocalin protein family: structure and function. Biochem J. 1996;318 (Pt 1):1–14. Epub 1996/08/15. ; PubMed Central PMCID: PMC1217580. 8761444 10.1042/bj3180001 PMC1217580

[26] Pelosi P. Perireceptor events in olfaction. J Neurobiol. 1996;30(1):3–19. Epub 1996/05/01. 10.1002/(SICI)1097-4695(199605)30:1&lt;3::AID-NEU2&gt;3.0.CO;2-A . 8727979

[27] Spinelli S, Ramoni R, Grolli S, Bonicel J, Cambillau C, Tegoni M. The structure of the monomeric porcine odorant binding protein sheds light on the domain swapping mechanism. Biochemistry. 1998;37(22):7913–8. Epub 1998/06/12. 10.1021/bi980179e . 9609684

[28] Staiano M, D'Auria S, Varriale A, Rossi M, Marabotti A, Fini C, et al Stability and dynamics of the porcine odorant-binding protein. Biochemistry. 2007;46(39):11120–7. Epub 2007/09/12. 10.1021/bi7008129 . 17845011

[29] Stepanenko OV, Marabotti A, Kuznetsova IM, Turoverov KK, Fini C, Varriale A, et al Hydrophobic interactions and ionic networks play an important role in thermal stability and denaturation mechanism of the porcine odorant-binding protein. Proteins. 2008;71(1):35–44. Epub 2007/10/09. 10.1002/prot.21658 . 17918730

[30] Burova TV, Choiset Y, Jankowski CK, Haertle T. Conformational stability and binding properties of porcine odorant binding protein. Biochemistry. 1999;38(45):15043–51. Epub 1999/11/11. doi: bi990769s. . 10555987 10.1021/bi990769s

[31] Paolini S, Tanfani F, Fini C, Bertoli E, Paolo P. Porcine odorant-binding protein: structural stability and ligand affinities measured by fourier-transform infrared spectroscopy and fluorescence spectroscopy. Biochim Biophys Acta. 1999;1431(1):179–88. Epub 1999/04/21. . 10209290 10.1016/s0167-4838(99)00037-0

[32] Kmiecik D, Albani JR. Effect of 1-aminoanthracene (1-AMA) binding on the structure of three lipocalin proteins, the dimeric beta lactoglobulin, the dimeric odorant binding protein and the monomeric alpha1-acid glycoprotein. Fluorescence spectra and lifetimes studies. J Fluoresc. 2010;20(5):973–83. Epub 2010/03/31. 10.1007/s10895-010-0643-z . 20352304

[33] Ramoni R, Vincent F, Ashcroft AE, Accornero P, Grolli S, Valencia C, et al Control of domain swapping in bovine odorant-binding protein. Biochem J. 2002;365(Pt 3):739–48. Epub 2002/04/05. 10.1042/BJ20011631 ; PubMed Central PMCID: PMC1222703. 11931632 PMC1222703

[34] Nespoulous C, Briand L, Delage MM, Tran V, Pernollet JC. Odorant binding and conformational changes of a rat odorant-binding protein. Chem Senses. 2004;29(3):189–98. Epub 2004/03/30. . 15047593 10.1093/chemse/bjh017

[35] Johansson JS, Manderson GA, Ramoni R, Grolli S, Eckenhoff RG. Binding of the volatile general anesthetics halothane and isoflurane to a mammalian beta-barrel protein. FEBS J. 2005;272(2):573–81. Epub 2005/01/19. 10.1111/j.1742-4658.2004.04500.x . 15654894

[36] Parisi M, Mazzini A, Sorbi RT, Ramoni R, Grolli S, Favilla R. Unfolding and refolding of porcine odorant binding protein in guanidinium hydrochloride: equilibrium studies at neutral pH. Biochim Biophys Acta. 2003;1652(2):115–25. Epub 2003/12/04. doi: S1570963903002760. . 14644047 10.1016/j.bbapap.2003.08.009

[37] Mikhailopulo KI, Serchenya TS, Kiseleva EP, Chernov YG, Tsvetkova TM, Kovganko NV, et al Interaction of molecules of the neonicotinoid imidacloprid and its structural analogs with human serum albumin. J Appl Spectrosc. 2008;75:857–63. Epub 31 January 2009. 10.1007/s10812-009-9120-3

[38] Lobel D, Strotmann J, Jacob M, Breer H. Identification of a third rat odorant-binding protein (OBP3). Chem Senses. 2001;26(6):673–80. Epub 2001/07/28. . 11473933 10.1093/chemse/26.6.673

[39] Meillour PN, Lagant P, Cornard JP, Brimau F, Le Danvic C, Vergoten G, et al Phenylalanine 35 and tyrosine 82 are involved in the uptake and release of ligand by porcine odorant-binding protein. Biochim Biophys Acta. 2009;1794(8):1142–50. Epub 2009/05/05. 10.1016/j.bbapap.2009.04.012 . 19410020

[40] Nespoulous C, Rofidal V, Sommerer N, Hem S, Rossignol M. Phosphoproteomic analysis reveals major default phosphorylation sites outside long intrinsically disordered regions of Arabidopsis plasma membrane proteins. Proteome Sci. 2012;10(1):62 Epub 2012/11/01. 10.1186/1477-5956-10-62 ; PubMed Central PMCID: PMC3537754. 23110452 PMC3537754

[41] Wang JC, Chu PY, Chen CM, Lin JH. idTarget: a web server for identifying protein targets of small chemical molecules with robust scoring functions and a divide-and-conquer docking approach. Nucleic Acids Res. 2012;40(Web Server issue):W393–9. Epub 2012/06/01. 10.1093/nar/gks496 ; PubMed Central PMCID: PMC3394295. 22649057 PMC3394295

[42] Chang DT, Oyang YJ, Lin JH. MEDock: a web server for efficient prediction of ligand binding sites based on a novel optimization algorithm. Nucleic Acids Res. 2005;33(Web Server issue):W233–8. Epub 2005/07/02. 10.1093/nar/gki586 ; PubMed Central PMCID: PMC1160262. 15991337 PMC1160262

[43] Morris GM, Huey R, Lindstrom W, Sanner MF, Belew RK, Goodsell DS, et al AutoDock4 and AutoDockTools4: Automated docking with selective receptor flexibility. J Comput Chem. 2009;30(16):2785–91. Epub 2009/04/29. 10.1002/jcc.21256 ; PubMed Central PMCID: PMC2760638. 19399780 PMC2760638

[44] Kim S, Thiessen PA, Bolton EE, Chen J, Fu G, Gindulyte A, et al PubChem Substance and Compound databases. Nucleic Acids Res. 2016;44(D1):D1202–13. Epub 2015/09/25. 10.1093/nar/gkv951 ; PubMed Central PMCID: PMC4702940. 26400175 PMC4702940

[45] Pettersen EF, Goddard TD, Huang CC, Couch GS, Greenblatt DM, Meng EC, et al UCSF Chimera: a visualization system for exploratory research and analysis. J Comput Chem. 2004;25(13):1605–12. Epub 2004/07/21. 10.1002/jcc.20084 . 15264254

[46] Berman HM, Westbrook J, Feng Z, Gilliland G, Bhat TN, Weissig H, et al The Protein Data Bank. Nucleic Acids Res. 2000;28(1):235–42. Epub 1999/12/11. ; PubMed Central PMCID: PMC102472. 10592235 10.1093/nar/28.1.235 PMC102472

[47] Goodsell DS, Morris G. M., Olson A. J. Automated docking of flexible ligands: applications of AutoDock. J Mol Recognit. 1996;9(1):1–5. Epub 1996/01/01. 10.1002/(SICI)1099-1352(199601)9:1&lt;1::AID-JMR241&gt;3.0.CO;2-6 . 8723313

[48] Vincent F, Spinelli S, Ramoni R, Grolli S, Pelosi P, Cambillau C, et al Complexes of porcine odorant binding protein with odorant molecules belonging to different chemical classes. J Mol Biol. 2000;300(1):127–39. Epub 2000/06/23. 10.1006/jmbi.2000.3820 . 10864504

[49] Vincent F, Ramoni R, Spinelli S, Grolli S, Tegoni M, Cambillau C. Crystal structures of bovine odorant-binding protein in complex with odorant molecules. Eur J Biochem. 2004;271(19):3832–42. Epub 2004/09/18. 10.1111/j.1432-1033.2004.04315.x . 15373829

[50] Gasteiger J, Marsili M. Iterative partial equalization of orbital electronegativity: a rapid access to atomic charges. Tetrahedron. 1980;36:3219–28. 10.1016/0040-4020(80)80168-2

[51] Hosainzadeh A, Gharanfoli M, Saberi M, Chamani J. Probing the interaction of human serum albumin with bilirubin in the presence of aspirin by multi-spectroscopic, molecular modeling and zeta potential techniques: insight on binary and ternary systems. J Biomol Struct Dyn. 2012;29(5):1013–50. Epub 2012/02/02. 10.1080/073911012010525029 . 22292958

[52] Laemmli UK. Cleavage of structural proteins during the assembly of the head of bacteriophage T4. Nature. 1970;227(5259):680–5. Epub 1970/08/15. . 5432063 10.1038/227680a0

[53] Moghaddam MM, Pirouzi M, Saberi MR, Chamani J. Comparison of the binding behavior of FCCP with HSA and HTF as determined by spectroscopic and molecular modeling techniques. Luminescence. 2014;29(4):314–31. Epub 2013/07/09. 10.1002/bio.2546 . 23832656

[54] Lakowicz JR. Principles of fluorescence spectroscopy 2rd ed. Kluwer Academic/Plenum: New York 1999.

[55] Albani JR. Principles and applications of fluorescence spectroscopy Blackwell l Science: Oxford 2007.

[56] Dallakyan S, Olson AJ. Small-molecule library screening by docking with PyRx. Methods Mol Biol. 2015;1263:243–50. Epub 2015/01/27. 10.1007/978-1-4939-2269-7_19 . 25618350

[57] EPA U. TRI Explorer 2014 National Analysis dataset. Estimation Program Interface (EPI) Suite Ver 41 US EPA, 2012. 2014;Available from: https://iaspub.epa.gov/triexplorer/tri_release.chemical.

[58] Halgren TA. MMFF VI. MMFF94s option for energy minimization studies. Journal of Computational Chemistry. 1999; 20(7):720–9. 10.1002/(SICI)1096-987X(199905)20:7<720::AID-JCC7>3.0.CO;2-X 34376030

[59] Khorsand Ahmadi S, Mahmoodian Moghadam M, Mokaberi P, Reza Saberi M, Chamani J. A comparison study of the interaction between beta-lactoglobulin and retinol at two different conditions: spectroscopic and molecular modeling approaches. J Biomol Struct Dyn. 2015;33(9):1880–98. Epub 2014/11/18. 10.1080/07391102.2014.977351 . 25402748

[60] Moosavi-Movahedi AA, Chamani J, Ghourchian H, Shafiey H, Sorenson CM, Sheibani N. Electrochemical evidence for the molten globule states of cytochrome c induced by N-alkyl sulfates at low concentrations. J Protein Chem. 2003;22(1):23–30. Epub 2003/05/13. . 12739895 10.1023/a:1023011609931

[61] Armbruster DA, Pry T. Limit of blank, limit of detection and limit of quantitation. Clin Biochem Rev. 2008;29 Suppl 1:S49–52. Epub 2008/10/15. ; PubMed Central PMCID: PMC2556583. 18852857 PMC2556583

