# Supplementary material for: The porcine odorant-binding protein as molecular probe for benzene detection
Source: PLoS One. 2018 Sep 5;13(9):e0202630. doi: 10.1371/journal.pone.0202630 (PMC6124761; doi:10.1371/journal.pone.0202630)
Supplement: S1 Table — (DOCX) [file pone.0202630.s001.docx]

| 1A18 | 1A2D | 1A3Y | 1A57 | 1AB0 | 1ACD | 1ADL | 1AEL | 1ALB | 1AQB | 1AVG | 1B0O | 1B4M | 1B56 |
| --- | --- | --- | --- | --- | --- | --- | --- | --- | --- | --- | --- | --- | --- |
| 1B8E | 1BBP | 1BEB | 1BJ7 | 1BLR | 1BM5 | 1BRP | 1BRQ | 1BSO | 1BSQ | 1BSY | 1BWY | 1CBI | 1CBQ |
| 1CBR | 1CBS | 1CJ5 | 1CRB | 1D2U | 1D3S | 1DC9 | 1DF3 | 1DFV | 1DV9 | 1DZJ | 1DZK | 1DZM | 1DZP |
| 1E00 | 1E02 | 1E06 | 1E5P | 1EAL | 1EII | 1EIO | 1EPA | 1EPB | 1EQD | 1ERB | 1ERX | 1EUO | 1EW3 |
| 1EXS | 1FDQ | 1FE3 | 1FEL | 1FEM | 1FEN | 1FTP | 1G5W | 1G74 | 1G7N | 1G85 | 1GGL | 1GKA | 1GM6 |
| 1GT1 | 1GT3 | 1GT4 | 1GT5 | 1GX8 | 1GX9 | 1GXA | 1H91 | 1HBP | 1HBQ | 1HMR | 1HMS | 1HMT | 1HN2 |
| 1HQP | 1I04 | 1I05 | 1I06 | 1I4U | 1ICM | 1ICN | 1IFB | 1IFC | 1IIU | 1IKE | 1IKJ | 1IW2 | 1JBH |
| 1JJJ | 1JJX | 1JV4 | 1JYD | 1JYJ | 1JZU | 1KGL | 1KOI | 1KQW | 1KQX | 1KT3 | 1KT4 | 1KT5 | 1KT6 |
| 1KT7 | 1KXO | 1KZW | 1KZX | 1L6M | 1LF7 | 1LFO | 1LIB | 1LIC | 1LID | 1LIE | 1LIF | 1LKE | 1LNM |
| 1LPJ | 1MDC | 1ML7 | 1MUP | 1MVG | 1MX7 | 1MX8 | 1N0S | 1NGL | 1NP1 | 1NP4 | 1O1U | 1O1V | 1O8V |
| 1OBP | 1OBQ | 1OBU | 1OEE | 1OEJ | 1OEK | 1OPA | 1OPB | 1P6P | 1PBO | 1PEE | 1PM1 | 1PMP | 1QAB |
| 1QFT | 1QFV | 1QG5 | 1QQS | 1QWD | 1QY0 | 1QY1 | 1QY2 | 1R0U | 1RBP | 1RLB | 1S2P | 1S44 | 1S7D |
| 1SA8 | 1SXU | 1SXW | 1SXX | 1SXY | 1SY0 | 1SY1 | 1SY2 | 1SY3 | 1T0V | 1T68 | 1T8V | 1TOU | 1TOW |
| 1TVQ | 1TW4 | 1TXL | 1U0X | 1U17 | 1U18 | 1URE | 1UZ2 | 1VPR | 1VYF | 1VYG | 1X71 | 1X89 | 1X8N |
| 1X8O | 1X8P | 1X8Q | 1X8U | 1XCA | 1XKI | 1YIV | 1YP6 | 1YP7 | 1YUP | 1YWA | 1YWB | 1YWC | 1YWD |
| 1Z24 | 1ZND | 1ZNE | 1ZNG | 1ZNH | 1ZNK | 1ZNL | 1ZRY | 2A0A | 2A13 | 2A2G | 2A2U | 2A3F | 2ACP |
| 2AH7 | 2AKQ | 2AL0 | 2ALL | 2AMM | 2ANS | 2ASN | 2AT0 | 2AT5 | 2AT6 | 2AT8 | 2BLG | 2CBR | 2CBS |
| 2DM5 | 2EU7 | 2F73 | 2FR2 | 2FR3 | 2FS6 | 2FS7 | 2FT9 | 2FTB | 2GC9 | 2GJ5 | 2GTF | 2HMB | 2HNX |
| 2HYS | 2IFB | 2JN3 | 2JOZ | 2JU3 | 2JU7 | 2JU8 | 2NNQ | 2NP1 | 2O62 | 2OFM | 2OZQ | 2Q2M | 2Q2P |
| 2Q39 | 2Q4N | 2Q9S | 2QM9 | 2R56 | 3BLG | 3C76 | 3C77 | 3C78 | 3CBS | 3EMM | 3IFB | 3NP1 | 4NP1 |
