# Supplementary material for: The porcine odorant-binding protein as molecular probe for benzene detection
Source: PLoS One. 2018 Sep 5;13(9):e0202630. doi: 10.1371/journal.pone.0202630 (PMC6124761; doi:10.1371/journal.pone.0202630)
Supplement: S2 Table — (DOCX) [file pone.0202630.s002.docx]

| **NAME** | **PUBCHEM ID** | **NAME** | **PUBCHEM ID** | **NAME** | **PUBCHEM ID** |
| --- | --- | --- | --- | --- | --- |
| 1,1,2,2-TETRACHLOROETHANE | 6591 | BUTYRALDEHYDE | 261 | METHOXYCHLOR | 4115 |
| 1,1,2-TRICHLOROETHANE | 6574 | CAPTAN | 8606 | METHYL HYDRAZINE | 6061 |
| 1,1-DIMETHYL HYDRAZINE | 5976 | CARBARYL | 6129 | METHYL IODIDE | 6328 |
| 1,2,4-TRICHLOROBENZENE | 13 | CARBON TETRACHLORIDE | 5943 | METHYL ISOBUTYL KETONE | 7909 |
| 1,2-BUTYLENE OXIDE | 7834 | CARBONIC ACID | 767 | METHYL ISOCYANATE | 12228 |
| 1,2-DIBROMO-3-CHLOROPROPANE | 7280 | CATECHOL | 289 | METHYL METHACRYLATE | 6658 |
| 1,2-DIBROMOETHANE | 11 | CHLORINE | 24526 | METHYL TERT-BUTYL ETHER | 15413 |
| 1,2-DICHLOROETHANE | 7839 | CHLOROACETIC ACID | 300 | M-XYLENE | 7929 |
| 1,2-DICHLORO-PROPANE | 10900 | CHLOROBENZENE | 7964 | N,N-DIMETHYLANILINE | 949 |
| 1,2-DIPHENYLHYDRAZINE | 31222 | CHLOROBENZILATE | 10522 | N,N-DIMETHYLFORMAMIDE | 6228 |
| 1,3-BUTADIENE | 7845 | CHLOROETHANE | 6337 | NAPHTHALENE | 931 |
| 1,3-DICHLOROPROPYLENE | 24726 | CHLOROFORM | 6212 | N-ETHYL-N-NITROSOUREA | 12967 |
| 1,4-DICHLOROBENZENE | 4685 | CHLOROMETHANE | 6327 | N-HEXANE | 8058 |
| 1,4-DIOXANE | 31275 | CHLOROMETHYL METHYL ETHER | 7864 | NITRIC ACID | 944 |
| 1-BUTANOL | 263 | CHLOROPRENE | 31369 | NITROBENZENE | 7416 |
| 2,4,5-TRICHLOROPHENOL | 7271 | CIS-3-CHLOROALLYL CHLORIDE | 24883 | N-NITROSO-N-METHYLUREA | 12699 |
| 2,4,6-TRICHLOROPHENOL | 6914 | CUMENE | 7406 | O-ANISIDINE | 7000 |
| 2,4-DIAMINOTOLUENE | 7261 | CYANAMIDE | 9864 | O-CRESOL | 335 |
| 2,4-DICHLOROPHENOXYACETIC ACID | 1486 | DIBENZOFURAN | 568 | O-TOLUIDINE | 7242 |
| 2,4-DINITROPHENOL | 1493 | DIBROMOMETHANE | 3024 | O-XYLENE | 7237 |
| 2,4-DINITROTOLUENE | 8461 | DIBUTYL PHTHALATE | 3026 | PARATHION | 991 |
| 2-ACETYLAMINOFLUORENE | 5897 | DICHLOROMETHANE | 6344 | P-CRESOL | 2879 |
| 2-NITROPROPANE | 398 | DIETHANOLAMINE | 8113 | PENTACHLOROBENZENE | 11855 |
| 3,3'-DICHLOROBENZIDINE | 7070 | DIETHYL SULFATE | 6163 | PENTACHLOROPHENOL | 992 |
| 3,3'-DIMETHOXYBENZIDINE | 8411 | DIMETHYL PHTHALATE | 8554 | PHENOL | 996 |
| 3,3'-DIMETHYLBENZIDINE | 8413 | DIMETHYL SULFATE | 6497 | PHENOLPHTHALEIN | 4764 |
| 3-AMINO-1,2,4-TRIAZOLE | 1639 | DIMETHYLAMINE | 674 | PHOSGENE | 6371 |
| 4-(2,4-DICHLOROPHENOXY)BUTANOIC ACID | 1489 | DIMETHYLCARBAMYL CHLORIDE | 6598 | PHOSPHINE | 24404 |
| 4,4'-METHYLENEBIS(2-CHLOROANILINE) | 7543 | EPICHLOROHYDRIN | 7835 | PHTHALIC ANHYDRIDE | 6811 |
| 4,4'-METHYLENEDIANILINE | 7577 | ETHYL ACRYLATE | 8821 | P-PHENYLENEDIAMINE | 7814 |
| 4,6-DINITRO-O-CRESOL | 10800 | ETHYLBENZENE | 7500 | PROPANE SULTONE | 14264 |
| 4-AMINOBIPHENYL | 7102 | ETHYLENE GLYCOL | 174 | PROPIONALDEHYDE | 527 |
| 4-NITROPHENOL | 980 | ETHYLENE OXIDE | 6354 | PROPOXUR | 4944 |
| ACETALDEHYDE | 177 | ETHYLENE THIOUREA | 2723650 | PROPYLENE OXIDE | 6378 |
| ACETAMIDE | 178 | ETHYLENEIMINE | 9033 | PROPYLENEIMINE | 6377 |
| ACETOPHENONE | 7410 | ETHYLIDENE DICHLORIDE | 6365 | PYRIDINE | 1049 |
| ACROLEIN | 7847 | FORMALDEHYDE | 712 | QUINOLINE | 7047 |
| ACRYLAMIDE | 6579 | FORMIC ACID | 284 | QUINONE | 4650 |
| ACRYLIC ACID | 6581 | HEPTACHLOR | 3589 | QUINTOZENE | 6720 |
| ACRYLONITRILE | 7855 | HEXACHLOROBENZENE | 8370 | STYRENE | 7501 |
| ALLYL CHLORIDE | 7850 | HEXACHLOROCYCLOPENTADIENE | 6478 | STYRENE OXIDE | 7276 |
| AMMONIA | 222 | HEXACHLOROETHANE | 6214 | TETRACHLOROETHYLENE | 31373 |
| ANILINE | 6115 | HEXACHLOROPHENE | 3598 | TOLUENE | 1140 |
| BENZENE | 241 | HYDRAZINE | 9321 | TOLUENE-2,4-DIISOCYANATE | 11443 |
| BENZIDINE | 7111 | HYDROCHLORIC ACID | 313 | TOXAPHENE | 5284469 |
| BENZOIC TRICHLORIDE | 7367 | HYDROGEN CYANIDE | 768 | TRICHLOROETHYLENE | 6575 |
| BENZYL CHLORIDE | 7503 | HYDROGEN FLUORIDE | 14917 | TRIETHYLAMINE | 8471 |
| BIPHENYL | 7095 | HYDROQUINONE | 785 | TRIFLURALIN | 5569 |
| BIS(2-CHLOROETHYL) ETHER | 8115 | ISOPROPANOL | 3776 | URETHANE | 5641 |
| BIS(CHLOROMETHYL) ETHER | 10967 | MALEIC ANHYDRIDE | 7923 | VINYL ACETATE | 7904 |
| BROMOFORM | 5558 | M-CRESOL | 342 | VINYL CHLORIDE | 6366 |
| BROMOMETHANE | 6323 | METHANOL | 887 | VINYLIDENE CHLORIDE | 6338 |
