# Supplementary figures and images for: The porcine odorant-binding protein as molecular probe for benzene detection
Source: PLoS One. 2018 Sep 5;13(9):e0202630. doi: 10.1371/journal.pone.0202630 (PMC6124761; doi:10.1371/journal.pone.0202630)

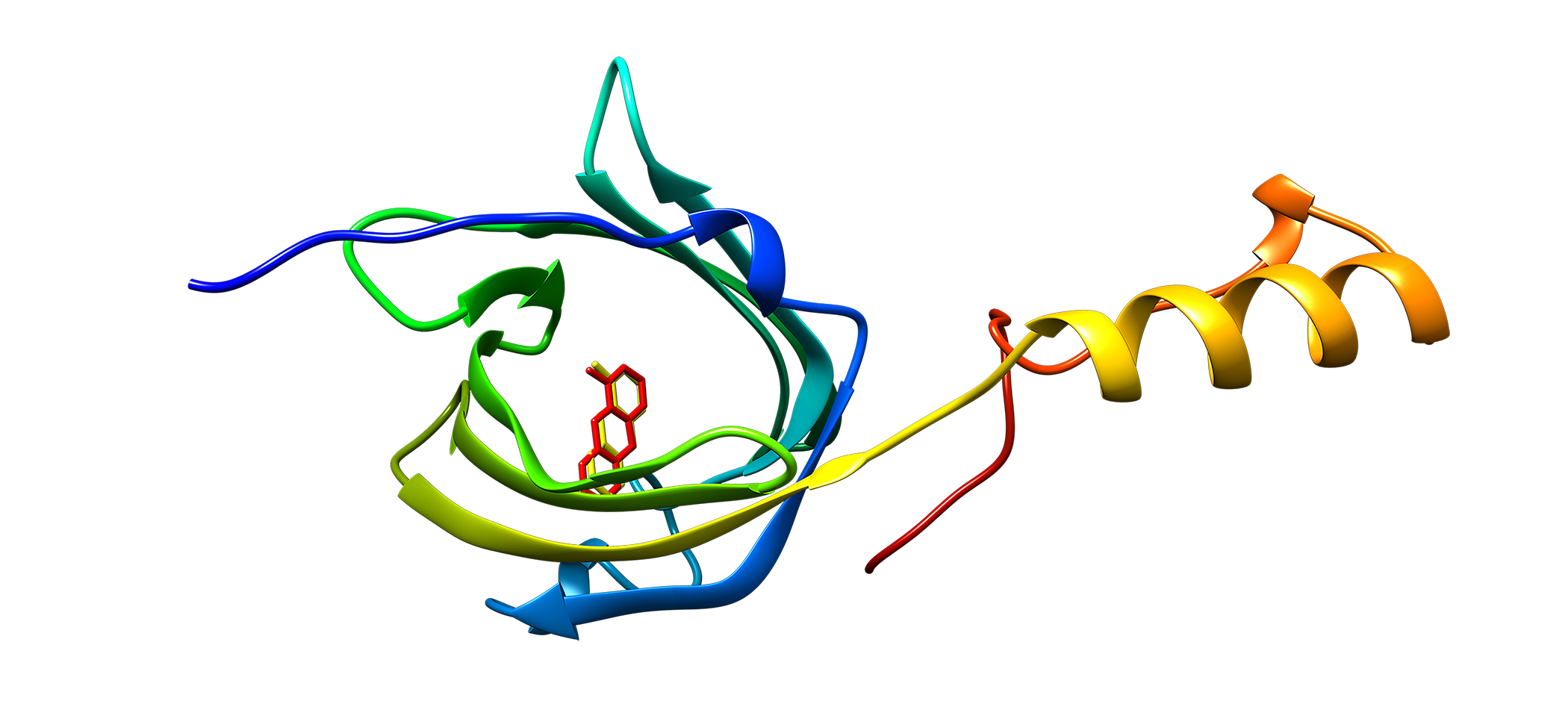

Supplement: S1 Fig — (TIF) [file pone.0202630.s003.tif]

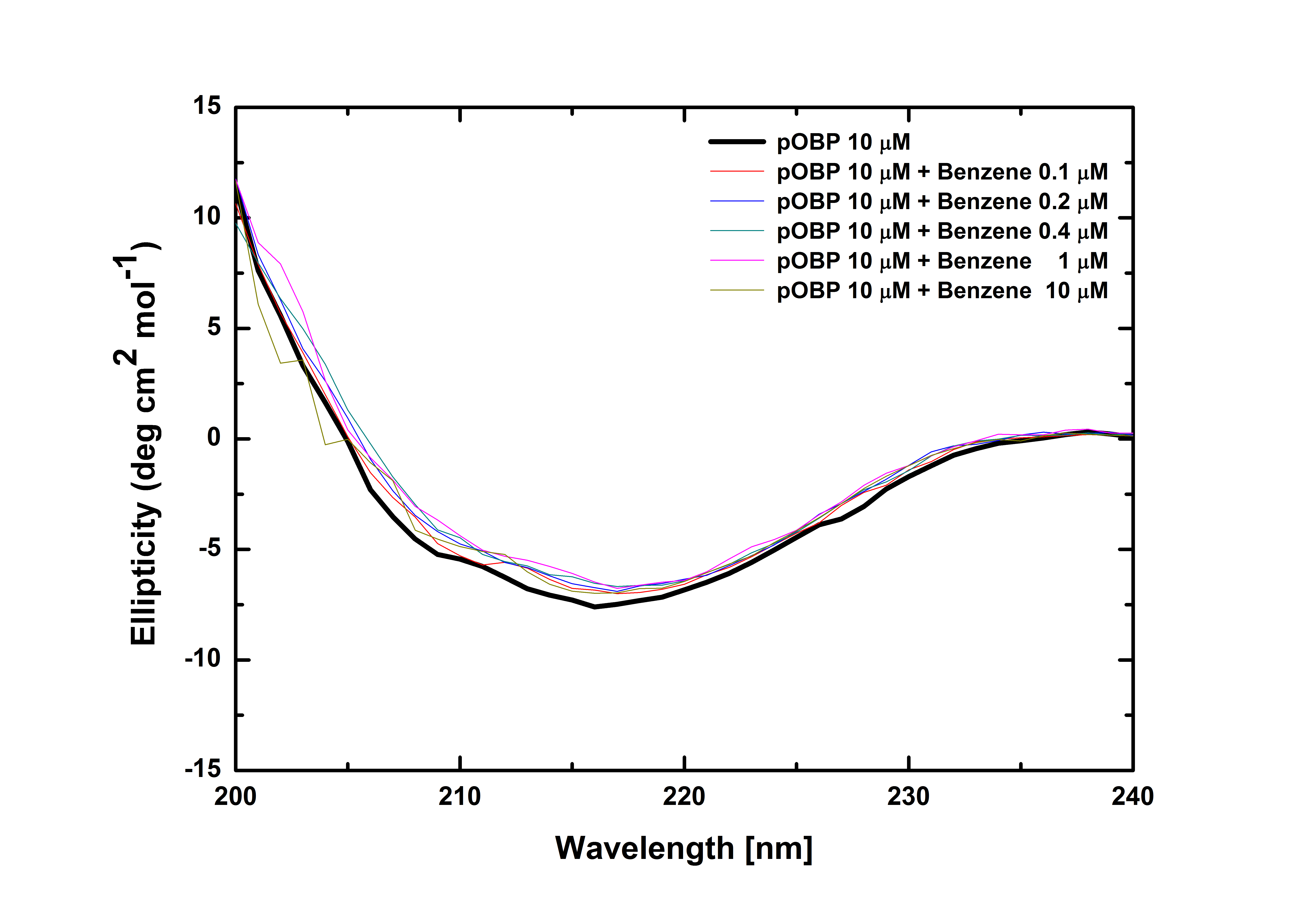

Supplement: S2 Fig — (TIF) [file pone.0202630.s004.tif]

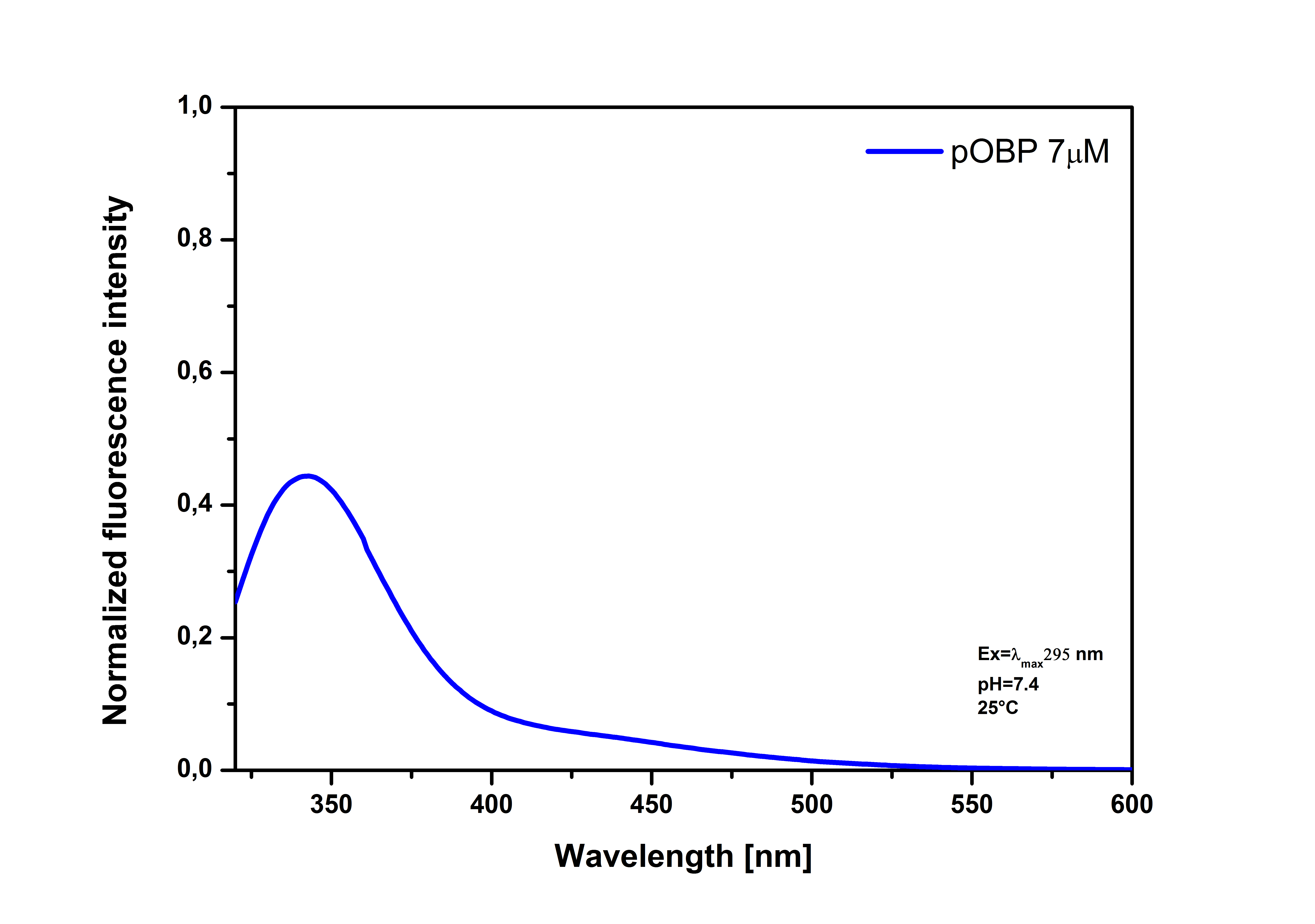

Supplement: S3 Fig — (TIF) [file pone.0202630.s005.tif]

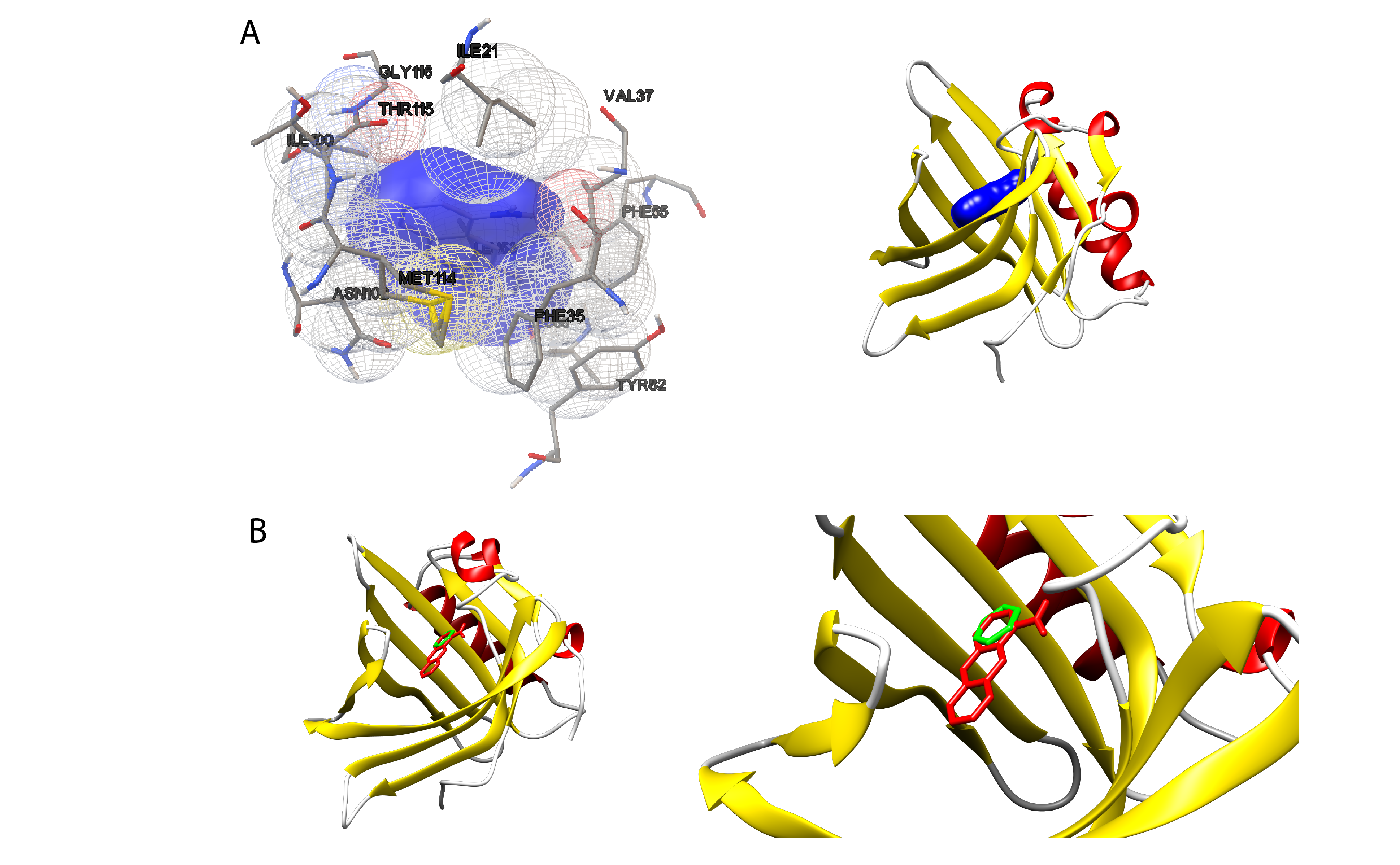

Supplement: S4 Fig — (A) On the left, a detail of pOBP binding site with highlighted amino acid residues involved in the interaction with the 1-AMA; on the right, the position of 1-AMA in the binding site of pOBP. (B) On the left, a superposition of benzene and 1-AMA docking pose into the binding site of pOBP, on the right, a particular where it is possible to appreciate the two molecules to share the protein binding site region. (TIF) [file pone.0202630.s006.tif]
